# Prevalence, Risk Factors, and Endoscopic Findings of *Helicobacter pylori* Infection Among Lebanese Patients Undergoing Gastroscopy: A Retrospective Study from a Single Tertiary Center

**DOI:** 10.3390/antibiotics14101013

**Published:** 2025-10-11

**Authors:** Rim Boutari, Nadeen Zayour, Ali Naji Hmedeh, Diana Khaled Bashashi, Fatima Assaf, Jana Al Tahan, Nancy Zrara, Nour Al-Mokdad, Omar Al Khatib, Abbas Zreik, Laura Akiki, Bilal Hoteit, Maha Hoteit, Zahra Sadek, Nikolaos Tzenios, Mahmoud Hallal

**Affiliations:** 1 Gastroenterology Department, Faculty of Medical Sciences, Lebanese University, Beirut P.O. Box 14-6573, Lebanon; reemboutari@gmail.com (R.B.); hmedehali4@gmail.com (A.N.H.); dianabashashi2017@gmail.com (D.K.B.); fatimaassaf891@gmail.com (F.A.); j.altahan@st.ul.edu.lb (J.A.T.); nancyzrara924@gmail.com (N.Z.); mkddnour876@gmail.com (N.A.-M.); o.alkhatib.1@st.ul.edu.lb (O.A.K.); abbaszrk97@gmail.com (A.Z.); laura_akiki@hotmail.com (L.A.); 2 Organized Research Unit, Al Zahraa Hospital University Medical Center (ZHUMC), Beirut P.O. Box 90-361, Lebanon; nadeenzayour6@gmail.com (N.Z.); m.hoteit@ul.edu.lb (M.H.); zahra.sadek@ul.edu.lb (Z.S.); 3 Badaro Endoscopic Center, Moarbes Hospital, Beirut P.O. Box 50-223, Lebanon; bilal.hotayt@hotmail.fr; 4 PHENOL Research Program, Faculty of Public Health, Section 1, Lebanese University, Beirut P.O. Box 6573, Lebanon; 5 Department of Primary Care and Population Health, University of Nicosia Medical School, Nicosia 2417, Cyprus; 6 INSPECT-LB (Institut National de Santé Publique, d’Épidémiologie Clinique et de Toxicologie-Liban), Beirut 1100, Lebanon; 7 Physiotherapy Department, Faculty of Public Health, Lebanese University, Beirut P.O. Box 6573-1, Lebanon; 8 Physical Therapy Department, Faculty of Public Health, Islamic University of Lebanon, Khaldeh P.O. Box 30014, Lebanon; 9 Faculty of Public Health, Charisma University, London EC1V 7QE, UK; 10 Gastroenterology and Hepatology Department, Al Zahraa Hospital University Medical Center (ZHUMC), Beirut P.O. Box 90-361, Lebanon

**Keywords:** *Helicobacter pylori*, *H. pylori* infection, gastroscopy, gastritis, duodenitis, intestinal metaplasia, Lebanon

## Abstract

(1) Background: Gastric cancer continues to pose a significant public health challenge, with its incidence influenced by various factors, including *Helicobacter pylori* (*H. pylori*) infection. In Lebanon, data on *H. pylori* prevalence and its associated risk factors remain limited. This study aimed to determine the prevalence of *H. pylori* infection among Lebanese outpatients presenting with gastrointestinal symptoms undergoing gastroscopy, to explore correlations between the infection and demographic and clinical variables, and to evaluate the prevalence of associated conditions such as gastritis, duodenitis, and intestinal metaplasia. (2) Methods: Using a retrospective design, data from 786 patients admitted at a hospital in Beirut over a three-year period were extracted from records. (3) Results: The prevalence of *H. pylori* infection was 29.6% despite 91.5% of patients showing signs of gastritis on endoscopy. The infection showed significant associations with erosive gastritis, non-erosive gastritis, mosaic gastritis, as well as with both erosive and non-erosive duodenitis. No significant relationships were observed between *H. pylori* and demographic factors, atrophic, or nodular gastritis. (4) Conclusions: These findings underscore the importance of targeted testing and early eradication of *H. pylori* to manage gastritis effectively and reduce the risk of progression to more serious gastric conditions in the Lebanese population.

## 1. Introduction

Gastric cancer remains a major global health challenge, ranking as the fifth-most commonly diagnosed cancer worldwide and the third leading cause of cancer-related deaths [1]. Despite advances in early detection and treatment, prognosis remains poor, especially in developing regions where diagnoses often occur at advanced stages [2]. In the Middle East, the incidence of gastric cancer varies across countries due to multiple contributing factors, including *Helicobacter pylori* (*H. pylori*) infection, dietary habits, smoking, and genetic predisposition, all of which contribute to regional disparities in disease burden [3]. Among the primary risk factors, *H. pylori* infection stands as the most well-established risk factor driving gastric carcinogenesis [4].

*H. pylori* is a Gram-negative, spiral-shaped, acid-resistant bacterium primarily colonizing the stomach’s pyloric region. The literature demonstrates its critical role in the development of gastric cancer, peptic ulcer disease, chronic gastritis, and mucosa-associated lymphoid tissue (MALT) lymphoma [5]. This infection can be spread by oral–oral or fecal–oral pathways and is commonly associated with poor sanitation, overcrowding, and the intake of contaminated food or water [6]. Although a significant number of carriers are asymptomatic, chronic infection provokes persistent gastric inflammation, causing mucosal damage, atrophic gastritis, and ultimately intestinal metaplasia, a recognized precursor for gastric cancer development [7]. The bacterium’s virulence factors interfere with host cellular signaling, promote epithelial cell proliferation, and inhibit apoptosis, thereby accelerating malignant transformation [8]. Additionally, alterations in the host immune response and gut microbiota composition have been implicated in *H. pylori*-induced carcinogenesis, underscoring the complexity of its pathogenic mechanisms [9]. These complications are associated with high morbidity and mortality, warranting its classification as a Group 1 carcinogen by the World Health Organization (WHO) [10].

Despite improvements in medical research and public health initiatives, *H. pylori* remains one of the most common chronic bacterial illnesses, posing a substantial challenge to healthcare systems [11]. Globally, the prevalence of *H. pylori* is estimated at 48.5%, with the highest rates reported in developing regions such as Africa (70.1%) and South America (69.4%). In the Middle East and North Africa (MENA) region, prevalence among adults ranges widely from 36.8 to 94%. In contrast, substantially lower prevalence rates are observed in developed areas like North America (37.1%) and Oceania (24.4%), largely influenced by factors such as socioeconomic conditions, hygiene practices, and healthcare accessibility [11,12]. In Lebanon, a reported prevalence of 52.4% has been observed in a single hospital-based investigation among symptomatic patients [13].

Given the high global burden of *H. pylori*, accurate and timely diagnosis is essential to prevent and manage associated diseases. There are several diagnostic techniques that range from non-invasive to invasive. Non-invasive methods such as serology, stool antigen tests, and the Carbon-13 (13C) urea breath test are commonly employed for initial screening [14]. However, invasive procedures like gastroscopy and biopsy remain the diagnostic gold standard, offering valuable information about the infection’s histological effects [10]. However, the effectiveness of eradication programs varies based on antibiotic resistance patterns, host factors, and regional prevalence, emphasizing the need for localized epidemiological studies [2].

Demographic factors and lifestyle habits, including smoking and alcohol consumption, have been shown to influence the colonization and persistence of *H. pylori* [15,16]. Notably, gender disparities have been observed, with some research suggesting that females may be more susceptible to the infection [16]. Age also appears to influence infection patterns, as the bacterium is typically acquired during childhood and can persist throughout life if left untreated [10]. A positive family history of gastric cancer has been identified as another potential risk factor, likely due to shared living environments and utensils that facilitate bacterial transmission [17,18]. Furthermore, the use of proton-pump inhibitors (PPIs) has been linked to lower infection rates, possibly because PPIs help suppress gastric acid secretion, thereby promoting mucosal healing and assisting in *H. pylori* eradication [19].

Therefore, understanding how *H. pylori* interacts with these risk factors is crucial for developing targeted diagnostics and personalized treatment strategies.

In Lebanon, both the prevalence and the associations between demographic characteristics, lifestyle habits, and *H. pylori* infection remain underexplored. Given the scarcity of data in the Lebanese population, this study seeks to address several key questions. Therefore, the objectives of this study are to: (1) assess the prevalence of *H. pylori* infection in patients presenting with gastrointestinal (GI) complaints who underwent gastroscopy at a tertiary care center in Lebanon; (2) explore associations between *H. pylori* infection and various demographic, lifestyle, and clinical factors; and (3) evaluate the occurrence and patterns of gastritis, duodenitis and intestinal metaplasia in relation to the bacterial infection.

## 2. Materials and Methods

### 2.1. Study Design

This observational cohort, single-center study was conducted retrospectively at Al-Zahraa Hospital University Medical Center (ZHUMC) in Beirut, Lebanon, by reviewing patient records from 1 January 2021, to 31 December 2023. The duration for the entire data collection phase, in which data was accessed for the research study purpose, was from 28 August 2024, to 30 December 2024.

### 2.2. Population and Sampling

The study population comprised adult Lebanese patients who underwent upper GI endoscopy at the ZHUMC endoscopy unit between January 2021 and December 2023 for various GI complaints. Eligible participants met the following inclusion criteria: (1) Lebanese nationality, (2) age between 18 and 65 years, (3) outpatient gastroscopy with biopsy collection during the procedure, and (4) no prior history of *H. pylori* infection or gastric surgery. Patients meeting these criteria were included in the study.

**Figure 1:**
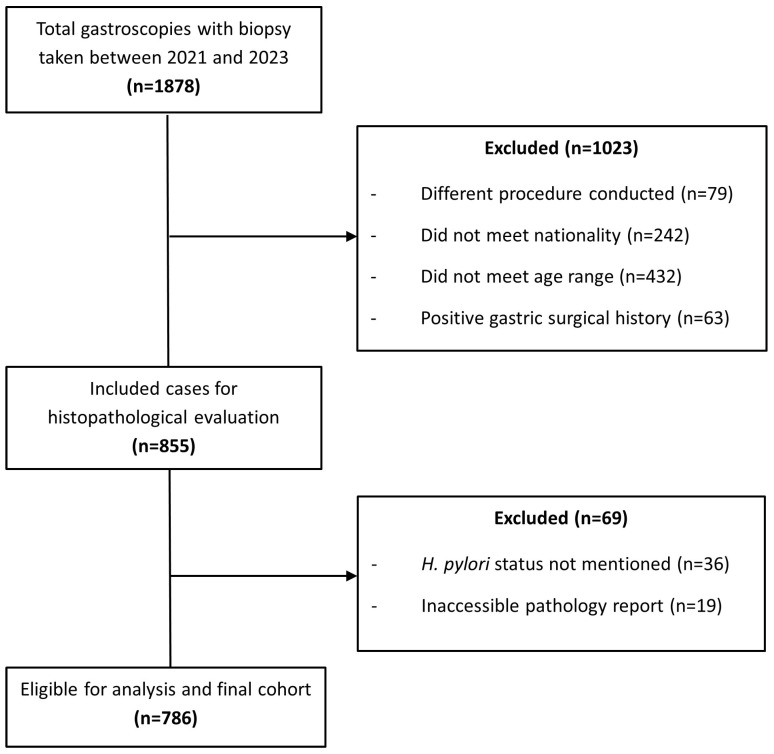
Flow diagram illustrating patient selection, inclusion and exclusion criteria for the cohort study.

Patients who underwent procedures other than gastroscopy, such as colonoscopy, endoscopic retrograde cholangiopancreatography (ERCP), or percutaneous endoscopic gastrostomy (PEG) were excluded. Patients were also excluded if diagnosed solely with conditions such as esophageal cancer, esophagitis, esophageal achalasia, esophageal stents, strictures, or varices. This exclusion is supported by evidence demonstrating that *H. pylori* predominantly colonizes the gastric mucosa and, in certain cases, the duodenal epithelium, while exhibiting minimal direct involvement in esophageal pathology. The bacterium’s adaptation to the acidic gastric environment, along with its specific affinity for gastric epithelial cells, underscores its limited pathogenic role in esophageal conditions [20]. Additionally, patients with missing or incomplete biopsy results, or those who had biopsies limited to non-gastric sites, such as the esophagus, were excluded. Finally, patients whose biopsy reports were inaccessible, or did not include *H. pylori* status, whether positive or negative, or who only had clot-based or rapid urease test results, were excluded. A flow diagram outlining inclusion and exclusion criteria and patient selection is presented in Figure 1.

### 2.3. Ethical Approval

The study was approved by the Institutional Review Board (IRB) at Al-Zahraa Hospital University Medical Center (approval number: 27/2024, approved on: 26 August 2024) and conducted according to the ethical standards of the Declaration of Helsinki. Written informed consent for file access was obtained from the attending physicians, and written informed consent was also secured from patients. Given the retrospective nature of the study, no additional patient contact was required. The data collection process was carried out by the study team in collaboration with a research assistant, ensuring accuracy and adherence to standardized protocols. All collected data were securely stored on a password-protected electronic drive in a restricted-access research unit office, ensuring compliance with data protection protocols. Access to the stored information was strictly limited to the research assistant in order to maintain confidentiality and prevent unauthorized retrieval. There was no attempt to identify any patients before or after the data collection process, and all patient data were anonymized to safeguard the privacy of the retrospectively gathered data. Once data collection was completed, the study team no longer had access to patient records.

### 2.4. Data Collection Procedures

#### 2.4.1. Endoscopy Logbooks

Data were initially extracted from the ZHUMC endoscopy unit’s logbooks. Initial patient identification was based on records of individuals who underwent gastroscopy, with or without concurrent colonoscopy, and included only those with biopsy sampling. A total of 1878 eligible patients were identified for further evaluation.

#### 2.4.2. Medical Records

Medical records of 1878 patients were reviewed retrospectively using the ZHUMC electronic medical record system. Data were sourced from two core gastroscopy documentation components:

- Admission documentation: this source provided demographic variables (age, sex, and marital status), anthropometric measurements (weight and height), clinical history (comorbidities, procedural history, and medication intake like PPIs, antibiotics, anticoagulants, etc.), and lifestyle habits (smoking and alcohol use).
- Procedural report: the gastroscopy report outlined procedural indications, endoscopic findings (mucosal abnormalities, ulcers, polyps, etc.), and biopsy sampling sites.

#### 2.4.3. Pathology Findings

**Figure 2:**
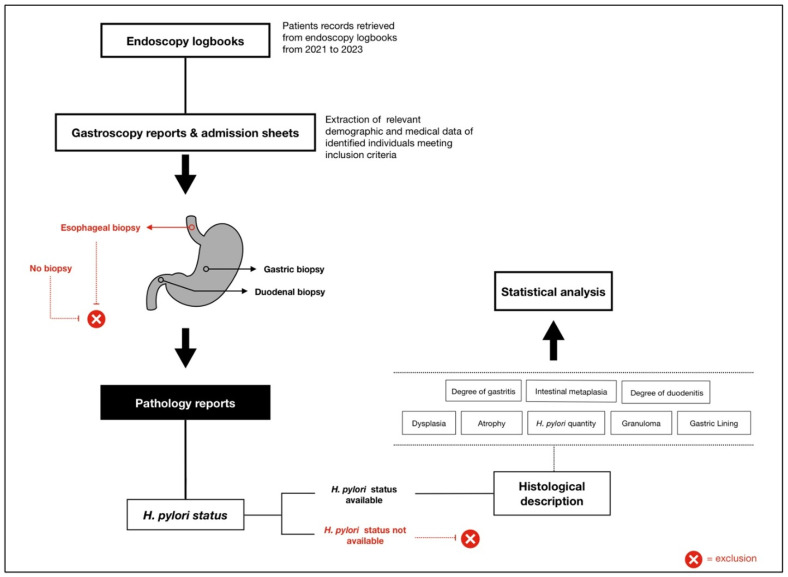
Overview of the study design and workflow for assessing *Helicobacter pylori* infection among Lebanese patients undergoing gastroscopy.

Final histopathological evaluation was performed for 855 subjects meeting criteria to confirm *H. pylori* infection through detailed biopsy report review. Histological findings were systematically recorded, including the presence of intestinal-type metaplasia, dysplasia, granuloma, and malignancy. The assessment also encompassed the severity and distribution of gastritis and/or duodenitis where relevant, ensuring mucosal pathology analysis. The presence of *H. pylori* was evaluated based on quantity and distribution of organisms within gastric or duodenal biopsies. The identified organisms were categorized into four groups: few crypts, several crypts, unspecified amount, or no evidence of the bacterial infection. The degree of inflammation was classified into six groups: mild, moderate, severe chronic inflammation; combined moderate/severe acute and chronic inflammation; or no inflammation. This detailed evaluation allowed for assessment of *H. pylori* presence and associated mucosal inflammation severity. After quality assessment and exclusion of non-qualifying cases, a final cohort of 786 records was retained for analysis (Figure 1). The study design and workflow, from patient retrieval from logbook review to data collection and analysis, is illustrated in Figure 2.

### 2.5. Statistical Analysis

Statistical analyses were performed using IBM SPSS Statistics, Version 22.0. Both descriptive and inferential statistical methods were applied to evaluate the prevalence of *H. pylori* infection among patients undergoing endoscopy over the past three years at ZHUMC and to examine associations with various variables. Descriptive statistics were used to characterize the sample’s demographic profile and to summarize medical and procedural histories, medication and supplement use, as well as biopsy sites and pathology findings. To assess the relationships between *H. pylori* infection and patient- and procedure-related factors, chi-square tests and regression analyses were conducted for categorical variables, while Mann–Whitney U tests were applied for continuous variables. Additional univariate analyses were performed to explore associations between patient-related factors and specific gastroduodenal conditions observed during gastroscopy, including atrophic gastritis, erosive gastritis, mosaic gastritis, nodular gastritis, non-erosive gastritis, erosive duodenitis, and non-erosive duodenitis. The relationship between *H. pylori* infection and each of these conditions was also assessed. A *p*-value < 0.05 was considered statistically significant for all tests.

## 3. Results

### 3.1. Demographic and Clinical Characteristics

The study included a final patient population of 786 individuals, with a mean age of 43.1 years, and a higher proportion of females (59.9%) compared to males (40.1%). The majority of patients were married (74.9%), while 22.5% were single and only 1.5% were divorced. Anthropometric data revealed a median weight of 72 kg and a median height of 167 cm. The median BMI was 25.5 kg/m^2^. Most patients had a normal BMI (45.9%) or were overweight (32.1%), while 17.4% were classified as obese. Regarding bowel habits, the majority of patients (68.2%) reported no abnormalities. However, 16.0% experienced constipation, 13.5% reported diarrhea, and 2.3% had alternating constipation and diarrhea. In terms of medical history, 85.8% of patients had between one and three conditions, while 2.9% had more than three. A significant portion (69.2%) had been previously hospitalized, and 62.7% had undergone one or two medical procedures. Only 7.5% reported undergoing a gastroscopy in the past five years. With respect to medication and supplement use, medication intake was evenly distributed among patients, whereas only 2.4% reported taking dietary supplements. As for lifestyle factors, a majority of patients were smokers (61.6%), while alcohol consumption was reported by just 6.7% (Table 1).

### 3.2. Medical History Profile

The data revealed a range of conditions among the study population, with GI disorders being the most prevalent, affecting 95.8% of patients. Other conditions such as hypertension (19.3%), diabetes (10.4%), heart disease (7.0%), and thyroid disorders (6.6%) were less frequent. The remaining conditions were relatively rare, each affecting fewer than 5% of patients, respectively. Patients may have more than one condition; thus, percentages do not sum to 100% (Figure S1).

### 3.3. Procedural History

The majority of patients (30.8%) had not undergone any previous procedures. Among those who had, the most common were gynecological procedures (15.8%), ear, nose, and throat (ENT) procedures (14.6%), cholecystectomy (9.3%), orthopedic procedures (9.3%), and endoscopic procedures (9.2%). Patients may have undergone more than one procedure; thus, percentages do not sum to 100% (Table S1).

### 3.4. Medication and Supplement Intake

Half of the patients (51.0%) were not taking any medications. Among those who were, the most commonly used were medications for cardiovascular conditions (18.6%), PPIs (12.5%), and antiplatelet agents (11.1%). Only a small proportion (0.8%) were taking antibiotics. Additionally, just 4.0% of patients reported using dietary supplements. Patients may be using more than one medication and/or supplement; thus, percentages do not sum to 100% (Figure S2).

### 3.5. Gastroscopy Procedural Characteristics

Table 1 presents the procedural characteristics among the study’s 786 patients, who underwent upper gastrointestinal evaluations. The procedures were nearly evenly divided between gastroscopy alone (43.8%) and combined gastroscopy with colonoscopy (56.2%). Procedures were performed relatively evenly across three years, 2021 (30.4%), 2022 (32.6%), and 2023 (37.0%), indicating consistent endoscopic activity over time. Epigastric pain was the predominant indication for endoscopy, reported in 85.8% of cases. Other common indications included gastroesophageal reflux disease (GERD) (16.0%) and diarrhea (11.1%). Alarm features such as anemia (9.3%), melena (3.3%), and weight loss (2.9%) were less frequently reported. The low rate of *H. pylori* serology-positive cases (0.3%) as an indication may reflect underutilization of non-invasive testing. It is important to note that patients may have more than one indication; thus, percentages do not sum to 100%. In terms of financial coverage, the majority of patients (74.4%) were self-payers, followed by those with medical insurance (11.1%). Notably, 6.5% of patients relied on donations, indicating a vulnerable subgroup within the population. This financial structure may influence both endoscopic referral and adherence to follow-up recommendations.

### 3.6. Gastroscopy Procedure Findings

**Figure 3:**
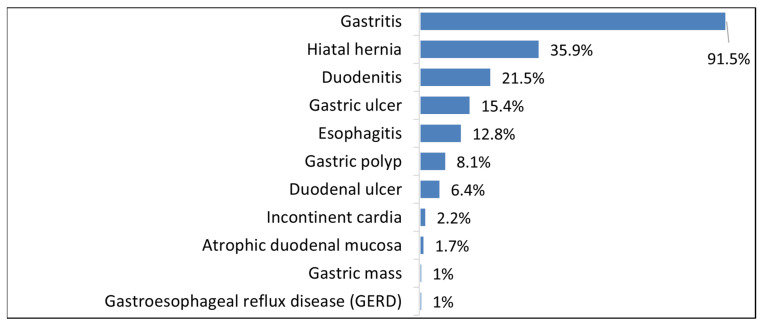
Distribution of gastrointestinal diagnoses identified through gastroscopy. Patients may present with multiple endoscopic findings; therefore, cumulative percentages exceed 100%. All findings are based on endoscopic observations, not histological analysis.

Gastroscopy findings in this cohort revealed a broad spectrum of upper gastrointestinal abnormalities, as detailed in Table S2. Patients may have more than one gastroscopy diagnosis; thus, the percentages do not add up to 100%. Gastritis was the most prevalent diagnosis, present in 91.5% of patients, followed by hiatal hernia (35.9%) and duodenitis (21.5%), the latter frequently occurring alongside gastritis, indicating a pattern of chronic upper GI disease. Gastric ulcers were observed in 15.4% of cases, possibly reflecting delayed diagnosis or insufficient management of underlying risk factors. Notably, some expected conditions were rarely identified: despite 16% of patients reporting symptoms suggestive of GERD, only one case of Barrett’s esophagus was detected, and just 1% were formally diagnosed with GERD, suggesting possible under-recognition of these conditions (Figure 3).

### 3.7. Endoscopic Interventions Performed During Gastroscopy

The data reflects a standardized approach to tissue sampling, with biopsies performed in all 786 cases (100%). However, this percentage is influenced by the study design, as patients without biopsies were excluded. This inclusion criterion ensures a focus on histopathological confirmation and may reflect either a protocol-driven diagnostic strategy or a high index of suspicion for conditions such as *H. pylori* infection, intestinal metaplasia, or malignancy. Interventional procedures were relatively uncommon. Polypectomy was the most common (1.7%), aligning with the 8.1% prevalence of gastric polyps, suggesting most were small and clinically insignificant. Use of clips (0.5%) and band ligation (0.4%) matched the number of patients with bleeding sources, gastric arteriovenous malformations (AVMs) and esophageal varices, indicating appropriate targeted treatment. Minimal use of argon plasma coagulation (0.1%) and hot forceps (0.4%) suggests limited need for hemostatic or ablative therapy. Overall, endoscopy in this cohort was largely diagnostic, with inflammatory findings predominating and few requiring therapeutic intervention. More than one endoscopic intervention may be performed; thus, percentages do not sum to 100%.

### 3.8. Source(s) of Biopsies Taken During Gastroscopy

Biopsy sampling was systematic, with gastric biopsies performed in 99.9% of cases, likely reflecting routine screening for *H. pylori*, intestinal metaplasia, or response to the high gastritis prevalence (91.5%). Duodenal biopsies were taken in 31.2% of cases, likely prompted by duodenitis (21.5%), suspected celiac disease, or duodenal ulcers (6.4%). This indicates a targeted approach to upper GI symptoms beyond the stomach. Esophageal biopsies were infrequent (3.3%) despite a 12.8% esophagitis rate, possibly due to visual diagnosis or low suspicion for Barrett’s or eosinophilic esophagitis. Lesion-targeted biopsies were rare, 0.5% for gastric polyps and 0.3% for ulcers, suggesting most were non-targeted unless clear abnormalities were present. Gastroesophageal junction (GEJ) sampling was minimal (0.1%), indicating low clinical concern for cardia intestinal metaplasia. Biopsy sampling could be from multiple sites; thus, percentages do not sum to 100%.

### 3.9. Histological Findings

#### 3.9.1. Lining Epithelium Types Identified

Endoscopic assessment showed that 71.9% of cases had non-ulcerated mucosa, indicating that most gastritis (91.5%) had not progressed to atrophy. Regenerating mucosa was seen in 17.9%, suggesting healing after *H. pylori* infection or inflammation. Hyperplastic changes (1.3%), eroded mucosa (0.8%), and ulceration (0.1%) were rare, aligning with the 15.4% gastric ulcer rate. About 9% showed non-ulcerated but abnormal mucosa, likely reflecting varying grades of gastritis. The low ulceration rate despite reported ulcers suggests many had healed or were localized. Overall, these findings indicate frequent but mostly non-severe gastric mucosal changes, underscoring the importance of *H. pylori* eradication and NSAID avoidance.

#### 3.9.2. Gastric Inflammation

**Figure 4:**
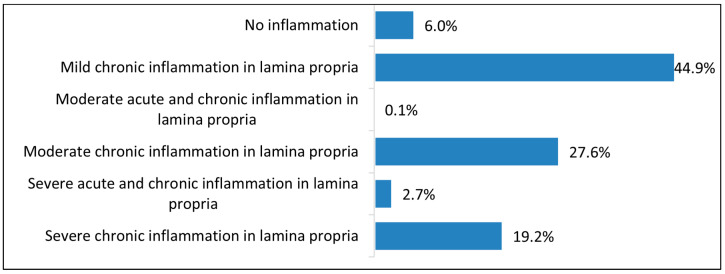
Distribution of inflammation severity in the lamina propria as determined by biopsy analysis.

Biopsy results revealed that only 6% of cases had no inflammation, suggesting normal mucosa or resolved inflammation. Most showed some inflammation: mild chronic inflammation was the most common (44.9%), typical of chronic *H. pylori* infection or NSAID use. Moderate chronic inflammation occurred in 27.6%, indicating more active or advanced disease. Severe chronic inflammation was less frequent (19.2%), suggesting early detection, effective treatment, or generally mild disease in this cohort (Figure 4).

#### 3.9.3. Prevalence of *H. pylori* and Other Histopathological Findings

The histopathological findings provide critical insights into gastric mucosal health and disease burden in this cohort. The vast majority of biopsies (99.2%) were classified as benign, with only 0.8% showing hyperplastic changes. The complete absence of malignancy (100% negative) is reassuring and may reflect a population with low cancer risk or effective early detection practices. *H. pylori* infection was identified in 29.6% of cases, most commonly with focal involvement, 20.1% limited to a few crypts and 9% involving several crypts, suggesting patchy rather than diffuse colonization. Precancerous changes were rare: intestinal metaplasia was observed in only 0.8% of cases, indicating limited progression toward malignant transformation, while granulomas were found in 0.3%, potentially reflecting chronic immune-mediated conditions such as Crohn’s disease or tuberculosis (Table 2).

### 3.10. Analysis of Risk Factors for H. pylori Infection

#### 3.10.1. Logistic Regression of Demographic and BMI Factors Predicting *H. pylori* Detection

A binary logistic regression was performed to assess whether age, gender, marital status, and BMI predicted *H. pylori* presence. The model was not statistically significant, χ^2^(6) = 6.02, *p* = 0.421, and explained only 1.1% of the variance in *H. pylori* detection (Nagelkerke R^2^ = 0.011). None of the predictors were statistically significant (all *p* > 0.19), although the constant was significant (*p* = 0.048), reflecting that the model overall predicts very low odds of *H. pylori* presence regardless of predictors. The model correctly classified 70.4% of cases overall but failed to correctly classify any positive cases. The logistic regression model did not significantly predict the presence of *H. pylori* organisms. None of the independent variables (age, gender, marital status, BMI) were significant predictors. The model explained very little variance (Nagelkerke R^2^ = 0.011) and failed to correctly identify any positive *H. pylori* cases. Each additional year of age increased the odds of *H. pylori* detection by only ~0.8% (OR = 1.01), but this effect was not significant, *p* = 0.23. Being male slightly reduced the odds of *H. pylori* compared to females (OR = 0.81), but this was not significant, *p* = 0.20. None of the marital categories significantly differed from widowed participants in odds of infection (all *p* > 0.27). BMI had almost no association with infection (OR ≈ 1.01, *p* = 0.73) (Table 3).

#### 3.10.2. Logistic Regression of Medical History Factors Predicting *H. pylori* Detection

A binary logistic regression was conducted to examine whether five dichotomous predictors (Crohn’s disease, Dyslipidemia, GERD, Hypertension, and None) predicted the presence of *H. pylori* organisms. The model was statistically significant compared with the null model, χ^2^(5) = 15.87, *p* = 0.007, but explained only 2.8% of the variance (Nagelkerke R^2^ = 0.028). Overall classification accuracy was 70.4%, but sensitivity was 0%, indicating the model failed to correctly identify any positive cases. Dyslipidemia showed a borderline association (B = 0.96, SE = 0.49, *p* = 0.051, OR = 2.61, 95% CI [0.99,6.83]) whereas GERD showed a non-significant trend (*p* = 0.082, OR = 1.48). The coefficient for Crohn’s disease could not be reliably estimated due to sparse data leading to quasi-complete separation (B ≈ 20, SE extremely large). No other predictors were statistically significant (Table 4).

#### 3.10.3. Logistic Regression of Medication Use Predicting *H. pylori* Detection

The logistic regression model including medication category, cardiovascular medication use, lipid-lowering therapy, and reporting no medications did not significantly predict the presence of *H. pylori*, χ^2^(5) = 9.09, *p* = 0.106. The model explained little variance (Nagelkerke R^2^ = 0.016) and had poor classification ability (overall accuracy = 70.4%, sensitivity = 0%). None of the predictors reached statistical significance, although the use of cardiovascular medications showed a non-significant trend toward increased odds of *H. pylori* infection (OR = 1.58, *p* = 0.077) (Table 5).

#### 3.10.4. Logistic Regression of Other Factors Predicting *H. pylori* Detection

The logistic regression model with gastroscopy history, smoking, alcohol use, guarantor type, and year of procedure showed a borderline overall fit, χ^2^(9) = 16.91, *p* = 0.050, explaining about 3% of the variance (Nagelkerke R^2^ = 0.030). Although the model was statistically marginal, it offered poor prediction (overall accuracy 70.7%, sensitivity only 4.3%). Among individual predictors, year of procedure (OR = 1.26, *p* = 0.026) and those who received donation to perform gastroscopy (OR = 1.88, *p* = 0.035) were associated with higher odds of *H. pylori* detection. Patients in the donation guarantor category had ~88% higher odds of *H. pylori* detection compared with the reference category. Year of procedure is a significant predictor (*p* = 0.026), each one-unit increase (later year) was associated with a 26% increase in odds of *H. pylori* detection (OR = 1.26). Other variables (gastroscopy within 5 years, smoking, alcohol use) were not significant predictors (Table 6).

### 3.11. Occurrence and Patterns of Gastric and Duodenal Diseases

The study population may have more than one gastric and duodenal disease; thus, the percentages do not add up to 100%. Erosive gastritis was the most common gastric finding (44.8%), which is usually linked to *H. pylori*, NSAIDs, alcohol, or bile reflux. Non-erosive gastritis occurred in 39.3%, indicating milder or early inflammation. Mosaic (9.7%) and nodular gastritis (2.8%) were less frequent, both associated with *H. pylori*. Atrophic gastritis was rare (1.9%), and varioliform gastritis very rare (0.1%). Unclassified cases due to normal findings made up 12.2%. Duodenitis was seen endoscopically in 21.5%, but histological confirmation was limited: erosive duodenitis in 14.1%, non-erosive 2.5%, atrophic 0.1%, and 78.5% with no pathological duodenitis, indicating partial agreement between endoscopy and biopsy.

#### 3.11.1. Atrophic Gastritis and Associated Factors

Atrophic gastritis was present in 1.9% (15/786) of patients. Age and sex differences were not significant, with more females affected (73.3%). Significant associations were found with autoimmune disease (6.7% vs. 0%; *p* = 0.019) and rheumatologic conditions (13.3% vs. 0.9%; *p* = 0.011). *H. pylori* presence showed no significant links, suggesting other causes beyond *H. pylori* (Table S3).

#### 3.11.2. Erosive Gastritis and Associated Factors

Erosive gastritis was seen in 44.8% (352/786), mostly in older patients (mean 44.17 years; *p* = 0.054). This pattern of gastritis was also more common in males (44.3% vs. 36.6%; *p* = 0.029) and was strongly associated with *H. pylori* presence (*p* < 0.001). Hence, *H. pylori* is the main correlate and the male predominance may reflect hormonal or behavioral factors while other factors such as lifestyle habits were not linked (Table S4).

#### 3.11.3. Mosaic Gastritis and Associated Factors

Mosaic gastritis affected 9.7% (76/786). Age was similar (*p* = 0.788), but males were more affected (51.3% vs. 38.9%; *p* = 0.035). No BMI difference was observed (*p* = 0.469). Mosaic gastritis was significantly associated with *H. pylori* infection (40.8% vs. 28.5%; *p* = 0.025) and less common in GERD (9.2% vs. 16.9%; *p* = 0.083). No links with diabetes, hypertension, or autoimmune diseases. Smoking and alcohol rates were similar (Table S5).

#### 3.11.4. Nodular Gastritis and Associated Factors

Nodular gastritis was rare (2.8%, 22/786). Patients with this pattern were slightly older (mean 47 vs. 43 years; *p* = 0.172) yet gender had no predominance. This pattern was significantly linked to dyslipidemia (18.2% vs. 4.3%; *p* = 0.017) and hypertension (40.9% vs. 18.7%; *p* = 0.009). In addition, patients with nodular gastritis were notably less likely to have other gastrointestinal disorders (59.1% vs. 80.2%; *p* = 0.015). *H. pylori* prevalence was lower among these diagnosed patients and did not reach significance (18.2% vs. 30.0%; *p* = 0.343) (Table S6).

#### 3.11.5. Non-Erosive Gastritis and Associated Factors

Non-erosive gastritis was identified in 309 of 786 patients (39.3%). These patients were significantly younger (mean age 41.6 vs. 44.2 years; *p* = 0.007) and more often female (66.0% vs. 56.0%; *p* = 0.005). They had a higher prevalence of underweight status (6.5% vs. 3.4%; *p* = 0.034) and lower obesity rates (13.6% vs. 19.9%). *H. pylori* infection was significantly less common (22.3% vs. 34.4%; *p* < 0.001), suggesting a different underlying pathophysiology compared to erosive gastritis. No significant differences were seen across other variable groups (Table S7).

#### 3.11.6. Erosive Duodenitis and Associated Factors

Erosive duodenitis showed significant male predominance (50.5% vs. 38.4%; *p* = 0.016) and higher alcohol use (13.5% vs. 5.6%; *p* = 0.002). Smoking trended higher but was not significant. No age difference (44.1 vs. 43.0 years), with more overweight in erosive cases (39.6% vs. 30.8%). *H. pylori* infection was more common among these patients (39.6% vs. 28.0%; *p* = 0.013). Neurological (5.4% vs. 1.8%; *p* = 0.018) and urological diseases (2.7% vs. 0.3%; *p* = 0.022) were also elevated and significantly linked with erosive duodenitis, while fewer erosive cases had other GI disorders (72.1% vs. 80.9%; *p* = 0.033) and more had no comorbidities (17.1% vs. 10.4%; *p* = 0.038). No significant links with GERD or metabolic/autoimmune conditions (Table S8).

#### 3.11.7. Non-Erosive Duodenitis and Associated Factors

Non-erosive duodenitis was identified in 20 of 786 patients (2.5%). These patients had a similar mean age (43.3 vs. 43.1 years) and gender distribution (45% male) compared to those without. No cases were underweight, but overweight (45% vs. 31.7%) and obesity (25% vs. 17.2%) were more common, suggesting a possible link to metabolic factors. Smoking was slightly more frequent (70% vs. 61.4%), while alcohol use was similar. Notably, *H. pylori* infection was significantly more prevalent in this group (50% vs. 29.1%; *p* = 0.043), which contrasts with its typical association with erosive forms. There was a complete absence of anemia, autoimmune, dyslipidemia, thyroid, and neurological disorders, and lower hypertension rates (5% vs. 19.7%). No cases of cancer, rheumatologic, psychiatric, or urological conditions were reported. This profile suggests that non-erosive duodenitis may represent a unique subgroup with strong *H. pylori* association and minimal comorbidity burden (Table S9).

## 4. Discussion

This retrospective analysis of 786 patients who underwent upper gastrointestinal endoscopy at a tertiary care center in Lebanon offers updated insights into the prevalence and characteristics of *H. pylori*. Based on histopathological biopsy results, the prevalence was 29.6%, which is notably lower than rates reported in previous Lebanese studies. For instance, a study by [14] reported an *H. pylori* prevalence ranging from 61.6% to 68.3% among adolescent and adult Lebanese individuals. The prevalence observed in our study also appears lower than that reported by [4], who found a prevalence of 52% in Tripoli, North of Lebanon. In contrast, the prevalence identified in our cohort was higher than that reported in a recent study by [21], which was conducted in another healthcare facility in Beirut. Compared to broader MENA region data, our results indicate a lower prevalence of *H. pylori* in Lebanon than that reported by [12], who observed prevalence rates ranging from 36.8% to 94% in adults. These discrepancies may be attributed to differences in study design, population characteristics including age distribution and variations in hygiene and socioeconomic status, diagnostic methods, temporal trends in infection rates [22].

Invasive procedures such as gastroscopy and biopsy remain the diagnostic gold standard for *H. pylori* detection, offering valuable insights into the infection’s histological impact—particularly in patients with atypical symptoms or elevated malignancy risk. Moreover, *H. pylori* eradication has emerged as a cornerstone of gastric cancer prevention strategies [10].

*H. pylori* has been strongly linked to conditions like non-ulcer dyspepsia, peptic ulcer disease, and chronic gastritis, all of which can manifest as epigastric discomfort or reflux-like symptoms [23]. However, in our study, despite a significant proportion of patients experiencing epigastric pain, only 29.6% tested positive for *H. pylori* infection. This suggests that epigastric pain is not specific to the infection and may be caused by other factors such as NSAID use, unhealthy eating habits, bile reflux, or functional dyspepsia [24,25]. Furthermore, non-invasive diagnostic methods, like serological testing, showed minimal use and very low detection (0.3%), suggesting a diagnostic pattern that favors invasive confirmation via endoscopy, similar to trends in some developed healthcare systems [26,27].

In terms of associated risk factors, our results revealed some unexpected patterns. Dyslipidemia was less common among *H. pylori*-positive individuals, possibly indicating a link between chronic infection and altered lipid metabolism through inflammatory or hormonal pathways [28,29,30]. It is noteworthy that a significant relationship was found between the year of the procedure and *H. pylori* positivity, suggesting potential temporal trends in infection rates or testing practices over the study period. Additionally, unlike prior studies, we found no significant associations with gender, older age, or high BMI [31,32,33], possibly due to variations in population demographics or lifestyle factors.

Proton-pump inhibitor (PPI) use has been linked to lower *H. pylori* infection rates, likely due to its ability to suppress gastric acid secretion, promote mucosal healing, and assist in bacterial eradication. However, excessive or prolonged PPI use may alter gastric pH, leading to complications such as vitamin deficiencies and increased risks of esophageal and gastric cancers [19]. Similarly, elevated body mass index (BMI) has been associated with increased susceptibility to *H. pylori* infection, potentially due to hormonal changes affecting metabolism and immune regulation [18].

The present study also examined *H. pylori*’s role in upper gastrointestinal disease. Duodenitis (21.5%), gastric ulcers (15.4%), and duodenal ulcers (6.4%) were among the notable findings. These results reaffirm the bacterium’s pathogenic role in disrupting mucosal defenses and increased gastric acid secretion [34]. Nonetheless, gastroscopy findings revealed a remarkably high prevalence of gastritis cases, affecting 91.5% of patients. However, only about one-third of those patients tested positive for *H. pylori* infection, suggesting multifactorial causes of mucosal inflammation. The discrepancy between the high rate of endoscopic gastritis (91.5%) and the lower histological *H. pylori* detection (29.6%) highlights the importance of biopsy in distinguishing infectious, autoimmune, and chemical causes of gastric inflammation. This dissociation also supports earlier findings that endoscopic features alone cannot reliably identify *H. pylori* infection [35]. Our results reinforce existing guidelines that recommend testing and eradicating *H. pylori* in patients with chronic inflammatory changes.

Histologically, nearly 45% of patients showed mild inflammation, and 47% had severe inflammation, consistent with the typical pattern of chronic *H. pylori* gastritis and mucosal immune activation reported in the literature [36]. As illustrated in Figure 4, the severity of lamina propria inflammation was significantly higher among *H. pylori*-positive individuals, reinforcing the bacterium’s role in driving mucosal immune response. This histological grading provides important clinical insight: patients with moderate to severe inflammation may be at increased risk for epithelial damage, atrophy, or progression to more serious gastric pathology. Thus, Figure 4 supports the use of biopsy not only for diagnosis but also for risk stratification and therapeutic planning. Atrophic gastritis was rare (1.9%) and not significantly associated with infection. Given that *H. pylori* is known to contribute to gastric atrophy through chronic inflammation and mucosal damage [37], other mechanisms such as autoimmune responses may play a role here [38]. This notion is supported by the association found between atrophic gastritis and immune-related diseases, consistent with previous studies linking autoimmune gastritis to systemic immune disorders [39,40]. It is also plausible that eradication therapy prevented progression to mucosal atrophy in previously infected individuals [41]. The low rates of metaplasia and absence of malignancy suggest limited short-term gastric cancer risk. Nonetheless, surveillance remains important, especially for those with metaplastic or atrophic changes. Erosive gastritis, present in nearly half the patients, was closely linked to *H. pylori* infection. Similar associations were found with mosaic gastritis and both forms of duodenitis. These reinforce the bacterium’s role in epithelial injury [42], consistent with the literature showing that *H. pylori* virulence factors like CagA and VacA (Cytotoxin-associated gene A and Vacuolating cytotoxin A) drive mucosal damage, inflammation, and impaired healing [43,44]. While other contributors like hormonal influences, NSAIDs, alcohol, and bile reflux remain relevant [45], our findings emphasize *H. pylori*’s prominent effect on erosive disease in this population. Interestingly, nodular gastritis, a relatively rare endoscopic finding, was associated with dyslipidemia and hypertension but not with *H. pylori*, which contrasts with the findings of [46]. Previous studies have indicated that metabolic syndrome can influence gastric mucosal changes through chronic low-grade inflammation, altered microcirculation, and immune dysregulation [47]. This suggests that in adults, nodularity may be more influenced by systemic metabolic or vascular factors. Among our patients, 39.3% had non-erosive gastritis. These individuals were generally younger, more often female, and more likely to be underweight, which aligns with the literature associating this gastritis type with functional GI symptoms and metabolic influences [48,49]. Notably, *H. pylori* was significantly less common in non-erosive cases, suggesting underlying mechanisms compared to erosive forms and supporting the need for individualized diagnosis and management.

One of the strengths of our study is the use of biopsy-based histological examination as the gold standard for diagnosing *H. pylori*, offering higher diagnostic accuracy compared to non-invasive methods [50]. Additionally, the presence of infection was confirmed through direct identification of the organism within gastric crypts and by grading the extent of colonization, in line with international diagnostic standards [51]. Despite these strengths, the study has limitations. As a retrospective, single-center study, it may suffer from selection bias and reduced generalizability. Efforts were made however to ensure that the inclusion criteria were highly specific, focusing on patients with biopsy-confirmed diagnoses and complete clinical records. Additionally, missing data led to the exclusion of certain files, including some *H. pylori*-positive cases, possibly underestimating the true prevalence. Lastly, while smoking and alcohol consumption were recorded and analyzed, other relevant clinical variables such as dietary habits were not consistently documented and therefore could not be assessed. This limitation prevented us from exploring associations with *H. pylori* infection as thoroughly as previous studies have done.

## 5. Conclusions

The findings of this study underscore the clinical relevance of *H. pylori* infection in Lebanon, revealing significant associations with multiple gastric and duodenal pathologies of different forms. These associations highlight the importance of targeted evaluation and monitoring, particularly in patients exhibiting precancerous conditions such as intestinal metaplasia or atrophy. While the immediate risk of gastric cancer appeared relatively low, long-term surveillance remains essential for individuals at elevated risk. Public health strategies should prioritize early detection and eradication efforts to reduce future complications. In light of the global emergence of multidrug-resistant (*MDR*) *H. pylori* strains, clinicians are encouraged to consider antibiogram-guided therapy when feasible, especially in cases of treatment failure or high local resistance rates [52,53]. Although treatment outcomes were beyond the scope of this retrospective study, our findings provide a foundation for future prospective research aimed at evaluating eradication success, resistance patterns, and long-term clinical benefits in the Lebanese population. Further research is warranted to monitor evolving epidemiological trends, explore regional disparities, assess environmental and host-related risk factors, and evaluate the effectiveness of different management approaches over time.

## Figures and Tables

**Table 1:** Demographic, clinical, procedural, and lifestyle characteristics of study participants.

Characteristics		*n* (%)
Age		43.15 ± 13.41
Gender	Male	315 (40.1%)
	Female	471 (59.9%)
Marital status	Single	177 (22.5%)
	Married	589 (74.9%)
	Divorced	12 (1.5%)
	Widowed	8 (1.0%)
Weight (kg)		72.10 ± 16.29
Height (cm)		167.81 ± 9.11
BMI (kg/m^2^)		25.50 ± 4.91
Body mass index (kg/m^2^)	Underweight (<18.5)	36 (4.6%)
	Normal weight (18.5–24.9)	361 (45.9%)
	Overweight (25.0–29.9)	252 (32.1%)
	Obese (≥30)	137 (17.4%)
Bowel movement	Constipation	126 (16.0%)
	Constipation and diarrhea	18 (2.3%)
	Diarrhea	106 (13.5%)
	No abnormality	536 (68.2%)
Medical history (as categories)	None	89 (11.3%)
	1 to 3 diseases	674 (85.8%)
	More than 3 diseases	23 (2.9%)
Previous hospitalization	Yes	544 (69.2%)
	No	242 (30.8%)
Number of procedures	None	242 (30.8%)
	1 to 2 procedures	493 (62.7%)
	3 or more procedures	51 (6.5%)
Gastroscopy within 5 years	Yes	59 (7.5%)
	No	727 (92.5%)
Procedure(s) conducted	Gastroscopy	344 (43.8%)
	Gastroscopy and colonoscopy	442 (56.2%)
Year of procedure	2021	239 (30.4%)
	2022	256 (32.6%)
	2023	291 (37.0%)
Indication for gastroscopy	Epigastric pain	674 (85.8%)
	Gastroesophageal reflux disease (GERD)	126 (16%)
	Diarrhea	87 (11.1%)
	Anemia	73 (9.3%)
	Nausea	48 (6.1%)
	Vomiting	48 (6.1%)
	Bloating	37 (4.7%)
	Melena	26 (3.3%)
	Weight loss	21 (2.7%)
	Dysphagia	18 (2.3%)
	FOBT positive or occult blood	18 (2.3%)
	Heartburn	12 (1.5%)
	Dyspepsia	10 (1.3%)
	Hematemesis	7 (0.9%)
	Belching	6 (0.8%)
	Vitamin B12 deficiency	6 (0.8%)
	Aphthous ulcers	3 (0.4%)
	History of gastric polyps	3 (0.4%)
	History of gastric ulcers	3 (0.4%)
	Pre-sleeve gastrectomy	3 (0.4%)
	Anorexia	2 (0.3%)
	Family history of gastric cancer	2 (0.3%)
	Rule out gastric cancer	2 (0.3%)
	*H. pylori* serology test positive	2 (0.3%)
	Family history of lynch syndrome	1 (0.1%)
	History of breast cancer	1 (0.1%)
Guarantor type	Donation	51 (6.5%)
	Hospital employees	14 (1.8%)
	Lebanese army and other forces	45 (5.7%)
	Lebanese municipalities	4 (0.5%)
	Medical insurance	87 (11.1%)
	Self-payer	585 (74.4%)
Medication intake	Yes	385 (49.0%)
	No	401 (51.0%)
Medication list	None	401 (51.0%)
	1 to 2 medications	308 (39.2%)
	3 or more medications	77 (9.8%)
	No	767 (97.6%)
Medications	None	401 (51.0%)
	Cardiovascular conditions medications	146 (18.6%)
	Other Medications	116 (14.8%)
	PPIs	98 (12.5%)
	Antiplatelets	87 (11.1%)
	Antidiabetics	58 (7.4%)
	Unknown/Unclear Medications	53 (6.7%)
	Thyroid medications	50 (6.4%)
	Lipid lowering medications	34 (4.3%)
	Painkillers	12 (1.5%)
	Antibiotics	6 (0.8%)
	Anticoagulants	5 (0.6%)
Supplement intake	Yes	19 (2.4%)
	No	767 (97.6%)
Supplements	None	767 (97.6%)
	Iron	8 (1.0%)
	Folic acid	7 (0.9%)
	Vitamin B12	4 (0.5%)
	Vitamin B6	3 (0.4%)
	Vitamin D	3 (0.4%)
	Magnesium	2 (0.3%)
	Calcium	1 (0.1%)
	Cobalamin	1 (0.1%)
	Herbal	1 (0.1%)
	Omega-3	1 (0.1%)
	Vitamin C	1 (0.1%)
Smoking	Yes	484 (61.6%)
	No	302 (38.4%)
Alcohol	Yes	53 (6.7%)
	No	733 (93.3%)

**Table 2:** Histopathological findings.

Finding		n (%)
Description	Benign	780 (99.2%)
	Hyperplastic	6 (0.8%)
Granuloma	Yes	2 (0.3%)
	No	784 (99.7%)
Metaplasia	Yes	6 (0.8%)
	No	780 (99.2%)
*H. pylori* organisms seen?	Yes	233 (29.6%)
	No	553 (70.4%)
*H. pylori* amount	*H. pylori* identified in few crypts	158 (20.1%)
	*H. pylori* identified in several crypts	71 (9.0%)
	Unspecified amount of *H. pylori*	4 (0.5%)
	No *H. pylori* organisms identified	553 (70.4%)

**Table 3:** Binary logistic regression analysis of age, gender, marital status, and BMI predicting *H. pylori* detection.

Predictor	B	SE	Wald	df	*p*	Exp (B)	95% CI Exp (B)	
							Lower	Upper
Age	0.008	0.007	1.45	1	0.228	1.01	0.99	1.02
Gender	−0.206	0.160	1.66	1	0.198	0.81	0.59	1.12
Marital status: Single (vs. Widowed)	1.20	1.09	1.21	1	0.271	3.33	0.39	28.45
Marital status: Married (vs. Widowed)	1.15	1.08	1.14	1	0.286	3.15	0.38	25.91
Marital status: Divorced (vs. Widowed)	0.40	1.32	0.09	1	0.762	1.49	0.11	19.93
BMI	0.006	0.017	0.12	1	0.731	1.01	0.94	1.04
Constant	−2.38	1.21	3.90	1	0.048	0.09		

**Table 4:** Logistic regression analysis of selected medical history predictors for *H. pylori* detection.

Predictor	B	SE	Wald	*p*	OR (Exp (B))	95% CI
Crohn’s disease	20.38	16,386.38	0.00	0.999	7.07 × 10^8^	–
Dyslipidemia	0.96	0.49	3.79	0.051	2.61	0.99–6.83
Gastroesophageal reflux disease (GERD)	0.40	0.23	3.03	0.082	1.48	0.95–2.32
Hypertension	0.34	0.34	0.97	0.324	1.40	0.72–2.76
None	−0.31	0.24	1.70	0.192	0.73	0.46–1.17
Constant	−22.53	16,386.38	0.00	0.999	0.00	–

**Table 5:** Logistic regression predicting *H. pylori* presence from medication categories.

Predictor	B	SE	Wald	*p*	OR (Exp (B))	95% CI for OR
Medication list overall			0.09	0.954	–	–
1 to 2 medications	−0.03	0.35	0.01	0.943	0.98	0.50–1.92
3 or more medications	−0.07	0.33	0.05	0.830	0.93	0.49–1.76
Cardiovascular medications	0.46	0.26	3.13	0.077	1.58	0.95–2.62
Lipid-lowering drugs	0.76	0.52	2.13	0.145	2.13	0.77–5.91
No medications	0.11	0.53	0.04	0.835	1.12	0.39–3.17
Constant	−2.04	0.72	7.99	0.005	0.13	–

**Table 6:** Binary logistic regression predicting *H. pylori* presence from gastroscopy history, smoking, alcohol use, guarantor type, and year of procedure.

Predictor	B	SE	Wald	*p*	OR (Exp (B))	95% CI for OR
Gastroscopy within 5 years	0.26	0.32	0.69	0.406	1.30	0.70–2.43
Smoking	−0.27	0.17	2.54	0.111	0.77	0.55–1.06
Alcohol	−0.14	0.31	0.21	0.643	0.87	0.47–1.60
Guarantor type overall	–	–	8.24	0.144	–	–
Donation	0.63	0.30	4.43	**0.035**	1.88	1.04–3.39
Hospital employees	0.19	0.57	0.11	0.742	1.21	0.39–3.71
Lebanese army and other forces	−0.42	0.37	1.28	0.257	0.66	0.32–1.36
Lebanese municipalities	1.17	1.02	1.32	0.251	3.21	0.44–23.45
Medical insurance	−0.18	0.27	0.45	0.505	0.84	0.50–1.41
Year of procedure	0.23	0.10	4.95	**0.026**	1.26	1.03–1.54
Constant	−462.96	207.61	4.97	0.026	~0.00	–

## Data Availability

The original contributions presented in this study are included in the article/Supplementary Material. Further inquiries can be directed to the corresponding author(s).

## Supplementary Materials

The following supporting information can be downloaded at: https://www.mdpi.com/article/10.3390/antibiotics14101013/s1, Figure S1: Medical history profile of the study population. Patients may have more than one condition; thus, percentages do not sum to 100%. FMF: familial Mediterranean fever, IBS: irritable bowel syndrome, PCOS: polycystic ovary syndrome; Table S1: Procedural history of the study population. Patients may have undergone more than one procedure; thus, percentages do not sum to 100%; Figure S2: Percentage of medication and supplement use by type among the study population. Patients may be using more than one medication and/or supplement; thus, percentages do not sum to 100%. PPIs: proton pump inhibitors; Table S2: Gastroscopy procedure findings among the study population. Patients may have more than one gastroscopy diagnosis; thus, the percentages do not add up to 100%; Table S3: Percent distribution and univariate analysis of factors associated with atrophic gastritis; Table S4: Percent distribution and univariate analysis of factors associated with erosive gastritis; Table S5: Percent distribution and univariate analysis of factors associated with mosaic gastritis; Table S6: Percent distribution and univariate analysis of factors associated with nodular gastritis; Table S7: Percent distribution and univariate analysis of factors associated with non-erosive gastritis; Table S8: Percent distribution and univariate analysis of factors associated with erosive duodenitis; Table S9: Percent distribution and univariate analysis of factors associated with non-erosive duodenitis.

## Abbreviations

The following abbreviations are used in this manuscript:

*H. pylori*	*Helicobacter pylori*
MALT	Mucosa-Associated Lymphoid Tissue
WHO	World Health Organization
MENA	Middle East and North Africa
PPIs	Proton Pump Inhibitors
BMI	Body Mass Index
GI	Gastrointestinal
ZHUMC	Al Zahraa Hospital University Medical Center
ERCP	Endoscopic Retrograde Cholangiopancreatography
PEG	Percutaneous Endoscopic Gastrostomy
IRB	Institutional Review Board
ENT	Ear, Nose, and Throat
GERD	Gastroesophageal Reflux Disease
FOBT	Fecal Occult Blood Test
AVMs	Arteriovenous Malformations
GEJ	Gastroesophageal Junction
FMF	Familial Mediterranean Fever
IBS	Irritable Bowel Syndrome
PCOS	Polycystic Ovary Syndrome
CagA	Cytotoxin-associated gene A
VacA	Vacuolating cytotoxin A
