# Supplementary material for: Prevalence, Risk Factors, and Endoscopic Findings of Helicobacter pylori Infection Among Lebanese Patients Undergoing Gastroscopy: A Retrospective Study from a Single Tertiary Center
Source: Antibiotics (Basel). 2025 Oct 11;14(10):1013. doi: 10.3390/antibiotics14101013 (PMC12561384; doi:10.3390/antibiotics14101013)
Supplement: Supplementary file 1 [file antibiotics-14-01013-s001.zip › Table_S1.pdf]

Table S1: Procedural history of the study population

Procedures previously conducted*	n (%)
None	242 (30.8%)
Gynecologic procedure	124 (15.8%)
Ear, nose, and throat (ENT) procedure	115 (14.6%)
Cholecystectomy	73 (9.3%)
Orthopedic procedure	73 (9.3%)
Endoscopic procedure	72 (9.2%)
Appendectomy	66 (8.4%)
Hernia repair	56 (7.1%)
Cardiac intervention	40 (5.1%)
Urological procedure	28 (3.6%)
Hemorrhoidectomy	27 (3.4%)
Cyst or abscess removal	23 (2.9%)
Unknown	21 (2.7%)
Breast surgery	20 (2.5%)
Plastic surgery	16 (2%)
Thyroidectomy	15 (1.9%)
Bowel surgery	14 (1.8%)
Eye surgery	12 (1.5%)
Anal fissure repair	8 (1%)
Vascular procedure	3 (0.4%)
Nephrological procedure	2 (0.3%)
Dermatologic procedure	1 (0.1%)
Esophagectomy	1 (0.1%)
Fistula repair	1 (0.1%)
Laparotomy	1 (0.1%)
Liver surgery	1 (0.1%)

*Patients may have undergone more than one procedure; thus, percentages do not sum to 100%
