# Supplementary material for: Prevalence, Risk Factors, and Endoscopic Findings of Helicobacter pylori Infection Among Lebanese Patients Undergoing Gastroscopy: A Retrospective Study from a Single Tertiary Center
Source: Antibiotics (Basel). 2025 Oct 11;14(10):1013. doi: 10.3390/antibiotics14101013 (PMC12561384; doi:10.3390/antibiotics14101013)
Supplement: Supplementary file 1 [file antibiotics-14-01013-s001.zip › Table_S2.pdf]

Table S2: Gastroscopy procedure findings among the study population

Diagnosis*	n (%)
Gastritis	719 (91.5%)
Hiatal hernia	282 (35.9%)
Duodenitis	169 (21.5%)
Gastric ulcer	121 (15.4%)
Esophagitis	101 (12.8%)
Gastric polyp	64 (8.1%)
Duodenal ulcer	50 (6.4%)
Incontinent cardia	17 (2.2%)
Atrophic duodenal mucosa	13 (1.7%)
Gastric mass	8 (1%)
Gastroesophageal reflux disease (GERD)	8 (1%)
Esophageal varices	4 (0.5%)
Gastric arteriovenous malformation (AVM)	4 (0.5%)
Gastric submucosal lesion	4 (0.5%)
Gastroesophageal junction (GEJ) ulcer	4 (0.5%)
Duodenal polyp	3 (0.4%)
Gastroparesis	3 (0.4%)
Esophageal ulcer	2 (0.3%)
Hypertrophic gastric folds	2 (0.3%)
Barret's esophagus	1 (0.1%)
Duodenal arteriovenous malformation (AVM)	1 (0.1%)
Duodenal mass	1 (0.1%)
Duodenal submucosal lesion	1 (0.1%)
Gastric varices	1 (0.1%)
Gastroesophageal junction (GEJ) lesion	1 (0.1%)
Lower esophageal candidiasis	1 (0.1%)
Normal gastroscopy	1 (0.1%)

* Patients may have more than one gastroscopy diagnosis; thus, the percentages do not add up to 100%.
