# Supplementary material for: Prevalence, Risk Factors, and Endoscopic Findings of Helicobacter pylori Infection Among Lebanese Patients Undergoing Gastroscopy: A Retrospective Study from a Single Tertiary Center
Source: Antibiotics (Basel). 2025 Oct 11;14(10):1013. doi: 10.3390/antibiotics14101013 (PMC12561384; doi:10.3390/antibiotics14101013)
Supplement: Supplementary file 1 [file antibiotics-14-01013-s001.zip › Table_S3.pdf]

Table S3: Percent distribution and univariate analysis of factors associated with atrophic gastritis

		Atrophic Gastritis			Univariate analysis
		**Overall n=786**	**Yes n=15**	**No n=771**	**P-value**
Age (Mean±Std)		43.15±13.4	43.93±13.2	43.09±13.4	0.417
Gender	Male	315 (40.1%)	4 (26.7%)	311 (40.3%)	0.426
	Female	471 (59.9%)	11 (73.3%)	460 (59.7%)	
Body mass index (kg/m^2)	Underweight (< 18.5)	36 (4.6%)	1 (6.7%)	35 (4.5%)	0.152
	Normal weight (18.5 - 24.9)	361 (45.9%)	10 (66.7%)	351 (45.5%)	
	Overweight (25.0 - 29.9)	252 (32.1%)	4 (26.7%)	248 (32.2%)	
	Obese (≥ 30)	137 (17.4%)	0 (0.0%)	137 (17.8%)	
Anemia	Yes	22 (2.8%)	1 (6.7%)	21 (2.7%)	0.349
	No	764 (97.2%)	14 (93.3%)	750 (97.3%)	
Autoimmune disease	Yes	1 (.1%)	1 (6.7%)	0 (0.0%)	**0.019**
	No	785 (99.9%)	14 (93.3%)	771 (100.0%)	
Bone disease	Yes	3 (.4%)	0 (0.0%)	3 (.4%)	1.000
	No	783 (99.6%)	15 (100.0%)	768 (99.6%)	
Cancer	Yes	15 (1.9%)	0 (0.0%)	15 (1.9%)	1.000
	No	771 (98.1%)	15 (100.0%)	756 (98.1%)	
Crohn's disease	Yes	6 (.8%)	0 (0.0%)	6 (.8%)	1.000
	No	780 (99.2%)	15 (100.0%)	765 (99.2%)	
Diabetes	Yes	82 (10.4%)	0 (0.0%)	82 (10.6%)	0.388
	No	704 (89.6%)	15 (100.0%)	689 (89.4%)	
Dyslipidemia	Yes	37 (4.7%)	0 (0.0%)	37 (4.8%)	1.000
	No	749 (95.3%)	15 (100.0%)	734 (95.2%)	
Familial Mediterranean fever (FMF)	Yes	3 (.4%)	0 (0.0%)	3 (.4%)	1.000
	No	783 (99.6%)	15 (100.0%)	768 (99.6%)	
Gastroesophageal reflux disease (GERD)	Yes	127 (16.2%)	3 (20.0%)	124 (16.1%)	0.721
	No	659 (83.8%)	12 (80.0%)	647 (83.9%)	
GI disorder	Yes	626 (79.6%)	13 (86.7%)	613 (79.5%)	0.748
	No	160 (20.4%)	2 (13.3%)	158 (20.5%)	
Heart disease	Yes	55 (7.0%)	0 (0.0%)	55 (7.1%)	0.616
	No	731 (93.0%)	15 (100.0%)	716 (92.9%)	
Hemorrhoids	Yes	1 (.1%)	0 (0.0%)	1 (.1%)	1.000
	No	785 (99.9%)	15 (100.0%)	770 (99.9%)	
Hypertension	Yes	152 (19.3%)	4 (26.7%)	148 (19.2%)	0.507
	No	634 (80.7%)	11 (73.3%)	623 (80.8%)	
Irritable bowel syndrome (IBS)	Yes	1 (.1%)	0 (0.0%)	1 (.1%)	1.000
	No	785 (99.9%)	15 (100.0%)	770 (99.9%)	

Kidney disease	Yes	7 (.9%)	0 (0.0%)	7 (.9%)	1.000
	No	779 (99.1%)	15 (100.0%)	764 (99.1%)	
Migraine	Yes	6 (.8%)	1 (6.7%)	5 (.6%)	0.110
	No	780 (99.2%)	14 (93.3%)	766 (99.4%)	
Neurological disease	Yes	18 (2.3%)	0 (0.0%)	18 (2.3%)	1.000
	No	768 (97.7%)	15 (100.0%)	753 (97.7%)	
Polycystic ovary syndrome (PCOS)	Yes	1 (.1%)	0 (0.0%)	1 (.1%)	1.000
	No	785 (99.9%)	15 (100.0%)	770 (99.9%)	
Peutz–Jeghers syndrome	Yes	0 (0.0%)	0 (0.0%)	0 (0.0%)	-
	No	786 (100.0%)	15 (100.0%)	771 (100.0%)	
Psoriasis	Yes	1 (.1%)	0 (0.0%)	1 (.1%)	1.000
	No	785 (99.9%)	15 (100.0%)	770 (99.9%)	
Psychiatric disorder	Yes	4 (.5%)	0 (0.0%)	4 (.5%)	1.000
	No	782 (99.5%)	15 (100.0%)	767 (99.5%)	
Respiratory disease	Yes	24 (3.1%)	0 (0.0%)	24 (3.1%)	1.000
	No	762 (96.9%)	15 (100.0%)	747 (96.9%)	
Rheumatological disease	Yes	9 (1.1%)	2 (13.3%)	7 (.9%)	**0.011**
	No	777 (98.9%)	13 (86.7%)	764 (99.1%)	
Thyroid disorder	Yes	52 (6.6%)	1 (6.7%)	51 (6.6%)	1.000
	No	734 (93.4%)	14 (93.3%)	720 (93.4%)	
Urological disease	Yes	5 (.6%)	0 (0.0%)	5 (.6%)	1.000
	No	781 (99.4%)	15 (100.0%)	766 (99.4%)	
Unknown	Yes	1 (.1%)	0 (0.0%)	1 (.1%)	1.000
	No	785 (99.9%)	15 (100.0%)	770 (99.9%)	
None	Yes	89 (11.3%)	1 (6.7%)	88 (11.4%)	1.000
	No	697 (88.7%)	14 (93.3%)	683 (88.6%)	
Smoker	Yes	484 (61.6%)	8 (53.3%)	476 (61.7%)	0.507
	No	302 (38.4%)	7 (46.7%)	295 (38.3%)	
Alcohol	Yes	53 (6.7%)	2 (13.3%)	51 (6.6%)	0.268
	No	733 (93.3%)	13 (86.7%)	720 (93.4%)	
H. pylori organisms seen?	Yes	233 (29.6%)	5 (33.3%)	228 (29.6%)	0.752
	No	553 (70.4%)	10 (66.7%)	543 (70.4%)	
