# Supplementary material for: Prevalence, Risk Factors, and Endoscopic Findings of Helicobacter pylori Infection Among Lebanese Patients Undergoing Gastroscopy: A Retrospective Study from a Single Tertiary Center
Source: Antibiotics (Basel). 2025 Oct 11;14(10):1013. doi: 10.3390/antibiotics14101013 (PMC12561384; doi:10.3390/antibiotics14101013)
Supplement: Supplementary file 1 [file antibiotics-14-01013-s001.zip › Table_S4.pdf]

Table S4: Percent distribution and univariate analysis of factors associated with erosive gastritis

		Erosive Gastritis			Univariate analysis
		Overall n=786	Yes n=352	No n=434	P-value
Age (Mean±Std)		43.15±13.4	44.17±13.0	42.32±13.7	0.054
Gender	Male	315 (40.1%)	156 (44.3%)	159 (36.6%)	**0.029**
	Female	471 (59.9%)	196 (55.7%)	275 (63.4%)	
Body mass index (kg/m^2)	Underweight (< 18.5)	36 (4.6%)	12 (3.4%)	24 (5.5%)	0.410
	Normal weight (18.5 - 24.9)	361 (45.9%)	160 (45.5%)	201 (46.3%)	
	Overweight (25.0 - 29.9)	252 (32.1%)	113 (32.1%)	139 (32.0%)	
	Obese (≥ 30)	137 (17.4%)	67 (19.0%)	70 (16.1%)	
Anemia	Yes	22 (2.8%)	8 (2.3%)	14 (3.2%)	0.420
	No	764 (97.2%)	344 (97.7%)	420 (96.8%)	
Autoimmune disease	Yes	1 (.1%)	0 (0.0%)	1 (.2%)	1.000
	No	785 (99.9%)	352 (100.0%)	433 (99.8%)	
Bone disease	Yes	3 (.4%)	1 (.3%)	2 (.5%)	1.000
	No	783 (99.6%)	351 (99.7%)	432 (99.5%)	
Cancer	Yes	15 (1.9%)	8 (2.3%)	7 (1.6%)	0.501
	No	771 (98.1%)	344 (97.7%)	427 (98.4%)	
Crohn's disease	Yes	6 (.8%)	3 (.9%)	3 (.7%)	1.000
	No	780 (99.2%)	349 (99.1%)	431 (99.3%)	
Diabetes	Yes	82 (10.4%)	40 (11.4%)	42 (9.7%)	0.441
	No	704 (89.6%)	312 (88.6%)	392 (90.3%)	
Dyslipidemia	Yes	37 (4.7%)	18 (5.1%)	19 (4.4%)	0.628
	No	749 (95.3%)	334 (94.9%)	415 (95.6%)	
Familial Mediterranean fever (FMF)	Yes	3 (.4%)	2 (.6%)	1 (.2%)	0.590
	No	783 (99.6%)	350 (99.4%)	433 (99.8%)	
Gastroesophageal reflux disease (GERD)	Yes	127 (16.2%)	56 (15.9%)	71 (16.4%)	0.865
	No	659 (83.8%)	296 (84.1%)	363 (83.6%)	
GI disorder	Yes	626 (79.6%)	273 (77.6%)	353 (81.3%)	0.191
	No	160 (20.4%)	79 (22.4%)	81 (18.7%)	
Heart disease	Yes	55 (7.0%)	28 (8.0%)	27 (6.2%)	0.344
	No	731 (93.0%)	324 (92.0%)	407 (93.8%)	
Hemorrhoids	Yes	1 (.1%)	0 (0.0%)	1 (.2%)	1.000
	No	785 (99.9%)	352 (100.0%)	433 (99.8%)	
Hypertension	Yes	152 (19.3%)	67 (19.0%)	85 (19.6%)	0.846
	No	634 (80.7%)	285 (81.0%)	349 (80.4%)	
Irritable bowel syndrome (IBS)	Yes	1 (.1%)	1 (.3%)	0 (0.0%)	0.448
	No	785 (99.9%)	351 (99.7%)	434 (100.0%)	
Kidney disease	Yes	7 (.9%)	4 (1.1%)	3 (.7%)	0.707
	No	779 (99.1%)	348 (98.9%)	431 (99.3%)	
Migraine	Yes	6 (.8%)	3 (.9%)	3 (.7%)	1.000

	No	780 (99.2%)	349 (99.1%)	431 (99.3%)	
Neurological disease	Yes	18 (2.3%)	11 (3.1%)	7 (1.6%)	0.159
	No	768 (97.7%)	341 (96.9%)	427 (98.4%)	
Polycystic ovary syndrome (PCOS)	Yes	1 (.1%)	0 (0.0%)	1 (.2%)	1.000
	No	785 (99.9%)	352 (100.0%)	433 (99.8%)	
Peutz–Jeghers syndrome	Yes	0 (0.0%)	0 (0.0%)	0 (0.0%)	-
	No	786 (100.0%)	352 (100.0%)	434 (100.0%)	
Psoriasis	Yes	1 (.1%)	1 (.3%)	0 (0.0%)	0.448
	No	785 (99.9%)	351 (99.7%)	434 (100.0%)	
Psychiatric disorder	Yes	4 (.5%)	2 (.6%)	2 (.5%)	1.000
	No	782 (99.5%)	350 (99.4%)	432 (99.5%)	
Respiratory disease	Yes	24 (3.1%)	14 (4.0%)	10 (2.3%)	0.175
	No	762 (96.9%)	338 (96.0%)	424 (97.7%)	
Rheumatological disease	Yes	9 (1.1%)	3 (.9%)	6 (1.4%)	0.738
	No	777 (98.9%)	349 (99.1%)	428 (98.6%)	
Thyroid disorder	Yes	52 (6.6%)	23 (6.5%)	29 (6.7%)	0.934
	No	734 (93.4%)	329 (93.5%)	405 (93.3%)	
Urological disease	Yes	5 (.6%)	3 (.9%)	2 (.5%)	0.661
	No	781 (99.4%)	349 (99.1%)	432 (99.5%)	
Unknown	Yes	1 (.1%)	1 (.3%)	0 (0.0%)	0.448
	No	785 (99.9%)	351 (99.7%)	434 (100.0%)	
None	Yes	89 (11.3%)	44 (12.5%)	45 (10.4%)	0.348
	No	697 (88.7%)	308 (87.5%)	389 (89.6%)	
Smoker	Yes	484 (61.6%)	223 (63.4%)	261 (60.1%)	0.357
	No	302 (38.4%)	129 (36.6%)	173 (39.9%)	
Alcohol	Yes	53 (6.7%)	26 (7.4%)	27 (6.2%)	0.517
	No	733 (93.3%)	326 (92.6%)	407 (93.8%)	
H. pylori organisms seen?	Yes	233 (29.6%)	127 (36.1%)	106 (24.4%)	**0.000**
	No	553 (70.4%)	225 (63.9%)	328 (75.6%)	
