# Supplementary material for: Prevalence, Risk Factors, and Endoscopic Findings of Helicobacter pylori Infection Among Lebanese Patients Undergoing Gastroscopy: A Retrospective Study from a Single Tertiary Center
Source: Antibiotics (Basel). 2025 Oct 11;14(10):1013. doi: 10.3390/antibiotics14101013 (PMC12561384; doi:10.3390/antibiotics14101013)
Supplement: Supplementary file 1 [file antibiotics-14-01013-s001.zip › Table_S5.pdf]

Table S5: Percent distribution and univariate analysis of factors associated with mosaic gastritis

		Mosaic Gastritis			Univariate analysis
		Overall n=786	Yes n=76	No n=710	P-value
Age (Mean±Std)		43.15±13.4	43.54±12.6	43.10±13.5	0.788
Gender	Male	315 (40.1%)	39 (51.3%)	276 (38.9%)	**0.035**
	Female	471 (59.9%)	37 (48.7%)	434 (61.1%)	
Body mass index (kg/m^2)	Underweight (< 18.5)	36 (4.6%)	2 (2.6%)	34 (4.8%)	0.469
	Normal weight (18.5 - 24.9)	361 (45.9%)	30 (39.5%)	331 (46.6%)	
	Overweight (25.0 - 29.9)	252 (32.1%)	29 (38.2%)	223 (31.4%)	
	Obese (≥ 30)	137 (17.4%)	15 (19.7%)	122 (17.2%)	
Anemia	Yes	22 (2.8%)	2 (2.6%)	20 (2.8%)	1.000
	No	764 (97.2%)	74 (97.4%)	690 (97.2%)	
Autoimmune disease	Yes	1 (.1%)	0 (0.0%)	1 (.1%)	1.000
	No	785 (99.9%)	76 (100.0%)	709 (99.9%)	
Bone disease	Yes	3 (.4%)	0 (0.0%)	3 (.4%)	1.000
	No	783 (99.6%)	76 (100.0%)	707 (99.6%)	
Cancer	Yes	15 (1.9%)	1 (1.3%)	14 (2.0%)	1.000
	No	771 (98.1%)	75 (98.7%)	696 (98.0%)	
Crohn's disease	Yes	6 (.8%)	0 (0.0%)	6 (.8%)	1.000
	No	780 (99.2%)	76 (100.0%)	704 (99.2%)	
Diabetes	Yes	82 (10.4%)	7 (9.2%)	75 (10.6%)	0.714
	No	704 (89.6%)	69 (90.8%)	635 (89.4%)	
Dyslipidemia	Yes	37 (4.7%)	4 (5.3%)	33 (4.6%)	0.775
	No	749 (95.3%)	72 (94.7%)	677 (95.4%)	
Familial Mediterranean fever (FMF)	Yes	3 (.4%)	0 (0.0%)	3 (.4%)	1.000
	No	783 (99.6%)	76 (100.0%)	707 (99.6%)	
Gastroesophageal reflux disease (GERD)	Yes	127 (16.2%)	7 (9.2%)	120 (16.9%)	0.083
	No	659 (83.8%)	69 (90.8%)	590 (83.1%)	
GI disorder	Yes	626 (79.6%)	64 (84.2%)	562 (79.2%)	0.298
	No	160 (20.4%)	12 (15.8%)	148 (20.8%)	
Heart disease	Yes	55 (7.0%)	3 (3.9%)	52 (7.3%)	0.349
	No	731 (93.0%)	73 (96.1%)	658 (92.7%)	
Hemorrhoids	Yes	1 (.1%)	0 (0.0%)	1 (.1%)	1.000
	No	785 (99.9%)	76 (100.0%)	709 (99.9%)	
Hypertension	Yes	152 (19.3%)	10 (13.2%)	142 (20.0%)	0.171
	No	634 (80.7%)	66 (86.8%)	568 (80.0%)	
Irritable bowel syndrome (IBS)	Yes	1 (.1%)	0 (0.0%)	1 (.1%)	1.000
	No	785 (99.9%)	76 (100.0%)	709 (99.9%)	
Kidney disease	Yes	7 (.9%)	1 (1.3%)	6 (.8%)	0.511
	No	779 (99.1%)	75 (98.7%)	704 (99.2%)	
Migraine	Yes	6 (.8%)	0 (0.0%)	6 (.8%)	1.000
	No	780 (99.2%)	76 (100.0%)	704 (99.2%)	
Neurological	Yes	18 (2.3%)	3 (3.9%)	15 (2.1%)	0.405

disease	No	768 (97.7%)	73 (96.1%)	695 (97.9%)	
Polycystic ovary syndrome (PCOS)	Yes	1 (.1%)	0 (0.0%)	1 (.1%)	1.000
	No	785 (99.9%)	76 (100.0%)	709 (99.9%)	
Peutz–Jeghers syndrome	Yes	0 (0.0%)	0 (0.0%)	0 (0.0%)	-
	No	786 (100.0%)	76 (100.0%)	710 (100.0%)	
Psoriasis	Yes	1 (.1%)	0 (0.0%)	1 (.1%)	1.000
	No	785 (99.9%)	76 (100.0%)	709 (99.9%)	
Psychiatric disorder	Yes	4 (.5%)	0 (0.0%)	4 (.6%)	1.000
	No	782 (99.5%)	76 (100.0%)	706 (99.4%)	
Respiratory disease	Yes	24 (3.1%)	1 (1.3%)	23 (3.2%)	0.721
	No	762 (96.9%)	75 (98.7%)	687 (96.8%)	
Rheumatological disease	Yes	9 (1.1%)	0 (0.0%)	9 (1.3%)	1.000
	No	777 (98.9%)	76 (100.0%)	701 (98.7%)	
Thyroid disorder	Yes	52 (6.6%)	5 (6.6%)	47 (6.6%)	0.989
	No	734 (93.4%)	71 (93.4%)	663 (93.4%)	
Urological disease	Yes	5 (.6%)	1 (1.3%)	4 (.6%)	0.399
	No	781 (99.4%)	75 (98.7%)	706 (99.4%)	
Unknown	Yes	1 (.1%)	0 (0.0%)	1 (.1%)	1.000
	No	785 (99.9%)	76 (100.0%)	709 (99.9%)	
None	Yes	89 (11.3%)	8 (10.5%)	81 (11.4%)	0.818
	No	697 (88.7%)	68 (89.5%)	629 (88.6%)	
Smoker	Yes	484 (61.6%)	49 (64.5%)	435 (61.3%)	0.585
	No	302 (38.4%)	27 (35.5%)	275 (38.7%)	
Alcohol	Yes	53 (6.7%)	5 (6.6%)	48 (6.8%)	0.952
	No	733 (93.3%)	71 (93.4%)	662 (93.2%)	
H. pylori organisms seen?	Yes	233 (29.6%)	31 (40.8%)	202 (28.5%)	**0.025**
	No	553 (70.4%)	45 (59.2%)	508 (71.5%)	
