# Supplementary material for: Prevalence, Risk Factors, and Endoscopic Findings of Helicobacter pylori Infection Among Lebanese Patients Undergoing Gastroscopy: A Retrospective Study from a Single Tertiary Center
Source: Antibiotics (Basel). 2025 Oct 11;14(10):1013. doi: 10.3390/antibiotics14101013 (PMC12561384; doi:10.3390/antibiotics14101013)
Supplement: Supplementary file 1 [file antibiotics-14-01013-s001.zip › Table_S6.pdf]

Table S6: Percent distribution and univariate analysis of factors associated with nodular gastritis

		Nodular Gastritis			Univariate analysis
		**Overall n=786**	**Yes n=22**	**No n=764**	**P-value**
Age (Mean±Std)		43.15±13.4	47.00±14.2	43.04±13.4	0.172
Gender	Male	315 (40.1%)	8 (36.4%)	307 (40.2%)	0.719
	Female	471 (59.9%)	14 (63.6%)	457 (59.8%)	
Body mass index (kg/m^2)	Underweight (< 18.5)	36 (4.6%)	2 (9.1%)	34 (4.5%)	0.479
	Normal weight (18.5 - 24.9)	361 (45.9%)	7 (31.8%)	354 (46.3%)	
	Overweight (25.0 - 29.9)	252 (32.1%)	8 (36.4%)	244 (31.9%)	
	Obese (≥ 30)	137 (17.4%)	5 (22.7%)	132 (17.3%)	
Anemia	Yes	22 (2.8%)	0 (0.0%)	22 (2.9%)	1.000
	No	764 (97.2%)	22 (100.0%)	742 (97.1%)	
Autoimmune disease	Yes	1 (.1%)	0 (0.0%)	1 (.1%)	1.000
	No	785 (99.9%)	22 (100.0%)	763 (99.9%)	
Bone disease	Yes	3 (.4%)	0 (0.0%)	3 (.4%)	1.000
	No	783 (99.6%)	22 (100.0%)	761 (99.6%)	
Cancer	Yes	15 (1.9%)	0 (0.0%)	15 (2.0%)	1.000
	No	771 (98.1%)	22 (100.0%)	749 (98.0%)	
Crohn's disease	Yes	6 (.8%)	0 (0.0%)	6 (.8%)	1.000
	No	780 (99.2%)	22 (100.0%)	758 (99.2%)	
Diabetes	Yes	82 (10.4%)	4 (18.2%)	78 (10.2%)	0.274
	No	704 (89.6%)	18 (81.8%)	686 (89.8%)	
Dyslipidemia	Yes	37 (4.7%)	4 (18.2%)	33 (4.3%)	**0.017**
	No	749 (95.3%)	18 (81.8%)	731 (95.7%)	
Familial Mediterranean fever (FMF)	Yes	3 (.4%)	0 (0.0%)	3 (.4%)	1.000
	No	783 (99.6%)	22 (100.0%)	761 (99.6%)	
Gastroesophageal reflux disease (GERD)	Yes	127 (16.2%)	4 (18.2%)	123 (16.1%)	0.769
	No	659 (83.8%)	18 (81.8%)	641 (83.9%)	
GI disorder	Yes	626 (79.6%)	13 (59.1%)	613 (80.2%)	**0.015**
	No	160 (20.4%)	9 (40.9%)	151 (19.8%)	
Heart disease	Yes	55 (7.0%)	2 (9.1%)	53 (6.9%)	0.662
	No	731 (93.0%)	20 (90.9%)	711 (93.1%)	
Hemorrhoids	Yes	1 (.1%)	0 (0.0%)	1 (.1%)	1.000
	No	785 (99.9%)	22 (100.0%)	763 (99.9%)	
Hypertension	Yes	152 (19.3%)	9 (40.9%)	143 (18.7%)	0.009
	No	634 (80.7%)	13 (59.1%)	621 (81.3%)	
Irritable bowel syndrome (IBS)	Yes	1 (.1%)	0 (0.0%)	1 (.1%)	1.000
	No	785 (99.9%)	22 (100.0%)	763 (99.9%)	
Kidney disease	Yes	7 (.9%)	0 (0.0%)	7 (.9%)	1.000
	No	779 (99.1%)	22 (100.0%)	757 (99.1%)	
Migraine	Yes	6 (.8%)	0 (0.0%)	6 (.8%)	1.000
	No	780 (99.2%)	22 (100.0%)	758 (99.2%)	
Neurological disease	Yes	18 (2.3%)	1 (4.5%)	17 (2.2%)	0.404
	No	768 (97.7%)	21 (95.5%)	747 (97.8%)	

Polycystic ovary syndrome (PCOS)	Yes	1 (.1%)	0 (0.0%)	1 (.1%)	1.000
	No	785 (99.9%)	22 (100.0%)	763 (99.9%)	
Peutz–Jeghers syndrome	Yes	0 (0.0%)	0 (0.0%)	0 (0.0%)	-
	No	786 (100.0%)	22 (100.0%)	764 (100.0%)	
Psoriasis	Yes	1 (.1%)	0 (0.0%)	1 (.1%)	1.000
	No	785 (99.9%)	22 (100.0%)	763 (99.9%)	
Psychiatric disorder	Yes	4 (.5%)	0 (0.0%)	4 (.5%)	1.000
	No	782 (99.5%)	22 (100.0%)	760 (99.5%)	
Respiratory disease	Yes	24 (3.1%)	1 (4.5%)	23 (3.0%)	0.499
	No	762 (96.9%)	21 (95.5%)	741 (97.0%)	
Rheumatological disease	Yes	9 (1.1%)	0 (0.0%)	9 (1.2%)	1.000
	No	777 (98.9%)	22 (100.0%)	755 (98.8%)	
Thyroid disorder	Yes	52 (6.6%)	2 (9.1%)	50 (6.5%)	0.652
	No	734 (93.4%)	20 (90.9%)	714 (93.5%)	
Urological disease	Yes	5 (.6%)	0 (0.0%)	5 (.7%)	1.000
	No	781 (99.4%)	22 (100.0%)	759 (99.3%)	
Unknown	Yes	1 (.1%)	0 (0.0%)	1 (.1%)	1.000
	No	785 (99.9%)	22 (100.0%)	763 (99.9%)	
None	Yes	89 (11.3%)	3 (13.6%)	86 (11.3%)	0.730
	No	697 (88.7%)	19 (86.4%)	678 (88.7%)	
Smoker	Yes	484 (61.6%)	15 (68.2%)	469 (61.4%)	0.518
	No	302 (38.4%)	7 (31.8%)	295 (38.6%)	
Alcohol	Yes	53 (6.7%)	3 (13.6%)	50 (6.5%)	0.180
	No	733 (93.3%)	19 (86.4%)	714 (93.5%)	
H. pylori organisms seen?	Yes	233 (29.6%)	4 (18.2%)	229 (30.0%)	0.343
	No	553 (70.4%)	18 (81.8%)	535 (70.0%)	
