# Supplementary material for: Prevalence, Risk Factors, and Endoscopic Findings of Helicobacter pylori Infection Among Lebanese Patients Undergoing Gastroscopy: A Retrospective Study from a Single Tertiary Center
Source: Antibiotics (Basel). 2025 Oct 11;14(10):1013. doi: 10.3390/antibiotics14101013 (PMC12561384; doi:10.3390/antibiotics14101013)
Supplement: Supplementary file 1 [file antibiotics-14-01013-s001.zip › Table_S7.pdf]

Table S7: Percent distribution and univariate analysis of factors associated with non-erosive gastritis

		Non-erosive Gastritis			Univariate analysis
		Overall<br>n=786	Yes<br>n=309	No<br>n=477	P-value
Age (Mean±Std)		43.15±13.4	41.55±13.7	44.18±13.2	**0.007**
Gender	Male	315 (40.1%)	105 (34.0%)	210 (44.0%)	**0.005**
	Female	471 (59.9%)	204 (66.0%)	267 (56.0%)	
Body mass index (kg/m^2)	Underweight (< 18.5)	36 (4.6%)	20 (6.5%)	16 (3.4%)	**0.034**
	Normal weight (18.5 - 24.9)	361 (45.9%)	143 (46.3%)	218 (45.7%)	
	Overweight (25.0 - 29.9)	252 (32.1%)	104 (33.7%)	148 (31.0%)	
	Obese (≥ 30)	137 (17.4%)	42 (13.6%)	95 (19.9%)	
Anemia	Yes	22 (2.8%)	10 (3.2%)	12 (2.5%)	0.550
	No	764 (97.2%)	299 (96.8%)	465 (97.5%)	
Autoimmune disease	Yes	1 (.1%)	0 (0.0%)	1 (.2%)	1.000
	No	785 (99.9%)	309 (100.0%)	476 (99.8%)	
Bone disease	Yes	3 (.4%)	2 (.6%)	1 (.2%)	0.565
	No	783 (99.6%)	307 (99.4%)	476 (99.8%)	
Cancer	Yes	15 (1.9%)	4 (1.3%)	11 (2.3%)	0.426
	No	771 (98.1%)	305 (98.7%)	466 (97.7%)	
Crohn's disease	Yes	6 (.8%)	3 (1.0%)	3 (.6%)	0.685
	No	780 (99.2%)	306 (99.0%)	474 (99.4%)	
Diabetes	Yes	82 (10.4%)	29 (9.4%)	53 (11.1%)	0.439
	No	704 (89.6%)	280 (90.6%)	424 (88.9%)	
Dyslipidemia	Yes	37 (4.7%)	14 (4.5%)	23 (4.8%)	0.851
	No	749 (95.3%)	295 (95.5%)	454 (95.2%)	
Familial Mediterranean fever (FMF)	Yes	3 (.4%)	1 (.3%)	2 (.4%)	1.000
	No	783 (99.6%)	308 (99.7%)	475 (99.6%)	
Gastroesophageal reflux disease (GERD)	Yes	127 (16.2%)	50 (16.2%)	77 (16.1%)	0.989
	No	659 (83.8%)	259 (83.8%)	400 (83.9%)	
GI disorder	Yes	626 (79.6%)	252 (81.6%)	374 (78.4%)	0.285
	No	160 (20.4%)	57 (18.4%)	103 (21.6%)	
Heart disease	Yes	55 (7.0%)	20 (6.5%)	35 (7.3%)	0.642
	No	731 (93.0%)	289 (93.5%)	442 (92.7%)	
Hemorrhoids	Yes	1 (.1%)	1 (.3%)	0 (0.0%)	0.393
	No	785 (99.9%)	308 (99.7%)	477 (100.0%)	
Hypertension	Yes	152 (19.3%)	61 (19.7%)	91 (19.1%)	0.818
	No	634 (80.7%)	248 (80.3%)	386 (80.9%)	
Irritable bowel syndrome (IBS)	Yes	1 (.1%)	0 (0.0%)	1 (.2%)	1.000
	No	785 (99.9%)	309 (100.0%)	476 (99.8%)	
Kidney disease	Yes	7 (.9%)	1 (.3%)	6 (1.3%)	0.255
	No	779 (99.1%)	308 (99.7%)	471 (98.7%)	
Migraine	Yes	6 (.8%)	2 (.6%)	4 (.8%)	1.000

	No	780 (99.2%)	307 (99.4%)	473 (99.2%)	
Neurological disease	Yes	18 (2.3%)	4 (1.3%)	14 (2.9%)	0.151
	No	768 (97.7%)	305 (98.7%)	463 (97.1%)	
Polycystic ovary syndrome (PCOS)	Yes	1 (.1%)	1 (.3%)	0 (0.0%)	0.393
	No	785 (99.9%)	308 (99.7%)	477 (100.0%)	
Peutz–Jeghers syndrome	Yes	0 (0.0%)	0 (0.0%)	0 (0.0%)	-
	No	786 (100.0%)	309 (100.0%)	477 (100.0%)	
Psoriasis	Yes	1 (.1%)	0 (0.0%)	1 (.2%)	1.000
	No	785 (99.9%)	309 (100.0%)	476 (99.8%)	
Psychiatric disorder	Yes	4 (.5%)	2 (.6%)	2 (.4%)	0.648
	No	782 (99.5%)	307 (99.4%)	475 (99.6%)	
Respiratory disease	Yes	24 (3.1%)	8 (2.6%)	16 (3.4%)	0.542
	No	762 (96.9%)	301 (97.4%)	461 (96.6%)	
Rheumatological disease	Yes	9 (1.1%)	6 (1.9%)	3 (.6%)	0.166
	No	777 (98.9%)	303 (98.1%)	474 (99.4%)	
Thyroid disorder	Yes	52 (6.6%)	21 (6.8%)	31 (6.5%)	0.871
	No	734 (93.4%)	288 (93.2%)	446 (93.5%)	
Urological disease	Yes	5 (.6%)	1 (.3%)	4 (.8%)	0.653
	No	781 (99.4%)	308 (99.7%)	473 (99.2%)	
Unknown	Yes	1 (.1%)	0 (0.0%)	1 (.2%)	1.000
	No	785 (99.9%)	309 (100.0%)	476 (99.8%)	
None	Yes	89 (11.3%)	29 (9.4%)	60 (12.6%)	0.168
	No	697 (88.7%)	280 (90.6%)	417 (87.4%)	
Smoker	Yes	484 (61.6%)	184 (59.5%)	300 (62.9%)	0.368
	No	302 (38.4%)	125 (40.5%)	177 (37.1%)	
Alcohol	Yes	53 (6.7%)	21 (6.8%)	32 (6.7%)	0.962
	No	733 (93.3%)	288 (93.2%)	445 (93.3%)	
H. pylori organisms seen?	Yes	233 (29.6%)	69 (22.3%)	164 (34.4%)	**0.000**
	No	553 (70.4%)	240 (77.7%)	313 (65.6%)	
