# Supplementary material for: Prevalence, Risk Factors, and Endoscopic Findings of Helicobacter pylori Infection Among Lebanese Patients Undergoing Gastroscopy: A Retrospective Study from a Single Tertiary Center
Source: Antibiotics (Basel). 2025 Oct 11;14(10):1013. doi: 10.3390/antibiotics14101013 (PMC12561384; doi:10.3390/antibiotics14101013)
Supplement: Supplementary file 1 [file antibiotics-14-01013-s001.zip › Table_S8.pdf]

Table S8: Percent distribution and univariate analysis of factors associated with erosive duodenitis

		Erosive Duodenitis			Univariate analysis
		Overall n=786	Yes n=111	No n=675	P-value
Age (Mean±Std)		43.15±13.4	44.13±13.6	42.99±13.4	0.406
Gender	Male	315 (40.1%)	56 (50.5%)	259 (38.4%)	**0.016**
	Female	471 (59.9%)	55 (49.5%)	416 (61.6%)	
Body mass index (kg/m^2)	Underweight (< 18.5)	36 (4.6%)	4 (3.6%)	32 (4.7%)	0.305
	Normal weight (18.5 - 24.9)	361 (45.9%)	48 (43.2%)	313 (46.4%)	
	Overweight (25.0 - 29.9)	252 (32.1%)	44 (39.6%)	208 (30.8%)	
	Obese (≥ 30)	137 (17.4%)	15 (13.5%)	122 (18.1%)	
Anemia	Yes	22 (2.8%)	3 (2.7%)	19 (2.8%)	1.000
	No	764 (97.2%)	108 (97.3%)	656 (97.2%)	
Autoimmune disease	Yes	1 (.1%)	0 (0.0%)	1 (.1%)	1.000
	No	785 (99.9%)	111 (100.0%)	674 (99.9%)	
Bone disease	Yes	3 (.4%)	0 (0.0%)	3 (.4%)	1.000
	No	783 (99.6%)	111 (100.0%)	672 (99.6%)	
Cancer	Yes	15 (1.9%)	3 (2.7%)	12 (1.8%)	0.458
	No	771 (98.1%)	108 (97.3%)	663 (98.2%)	
Crohn's disease	Yes	6 (.8%)	1 (.9%)	5 (.7%)	0.600
	No	780 (99.2%)	110 (99.1%)	670 (99.3%)	
Diabetes	Yes	82 (10.4%)	10 (9.0%)	72 (10.7%)	0.596
	No	704 (89.6%)	101 (91.0%)	603 (89.3%)	
Dyslipidemia	Yes	37 (4.7%)	4 (3.6%)	33 (4.9%)	0.808
	No	749 (95.3%)	107 (96.4%)	642 (95.1%)	
Familial Mediterranean fever (FMF)	Yes	3 (.4%)	1 (.9%)	2 (.3%)	0.367
	No	783 (99.6%)	110 (99.1%)	673 (99.7%)	
Gastroesophageal reflux disease (GERD)	Yes	127 (16.2%)	19 (17.1%)	108 (16.0%)	0.767
	No	659 (83.8%)	92 (82.9%)	567 (84.0%)	
GI disorder	Yes	626 (79.6%)	80 (72.1%)	546 (80.9%)	**0.033**
	No	160 (20.4%)	31 (27.9%)	129 (19.1%)	
Heart disease	Yes	55 (7.0%)	10 (9.0%)	45 (6.7%)	0.370
	No	731 (93.0%)	101 (91.0%)	630 (93.3%)	
Hemorrhoids	Yes	1 (.1%)	0 (0.0%)	1 (.1%)	1.000
	No	785 (99.9%)	111 (100.0%)	674 (99.9%)	
Hypertension	Yes	152 (19.3%)	20 (18.0%)	132 (19.6%)	0.704
	No	634 (80.7%)	91 (82.0%)	543 (80.4%)	
Irritable bowel syndrome (IBS)	Yes	1 (.1%)	0 (0.0%)	1 (.1%)	1.000
	No	785 (99.9%)	111 (100.0%)	674 (99.9%)	
Kidney disease	Yes	7 (.9%)	1 (.9%)	6 (.9%)	1.000
	No	779 (99.1%)	110 (99.1%)	669 (99.1%)	
Migraine	Yes	6 (.8%)	1 (.9%)	5 (.7%)	0.600
	No	780 (99.2%)	110 (99.1%)	670 (99.3%)	
Neurological	Yes	18 (2.3%)	6 (5.4%)	12 (1.8%)	**0.018**

disease	No	768 (97.7%)	105 (94.6%)	663 (98.2%)	
Polycystic ovary syndrome (PCOS)	Yes	1 (.1%)	0 (0.0%)	1 (.1%)	1.000
	No	785 (99.9%)	111 (100.0%)	674 (99.9%)	
Peutz–Jeghers syndrome	Yes	0 (0.0%)	0 (0.0%)	0 (0.0%)	-
	No	786 (100.0%)	111 (100.0%)	675 (100.0%)	
Psoriasis	Yes	1 (.1%)	0 (0.0%)	1 (.1%)	1.000
	No	785 (99.9%)	111 (100.0%)	674 (99.9%)	
Psychiatric disorder	Yes	4 (.5%)	0 (0.0%)	4 (.6%)	1.000
	No	782 (99.5%)	111 (100.0%)	671 (99.4%)	
Respiratory disease	Yes	24 (3.1%)	1 (.9%)	23 (3.4%)	0.233
	No	762 (96.9%)	110 (99.1%)	652 (96.6%)	
Rheumatological disease	Yes	9 (1.1%)	1 (.9%)	8 (1.2%)	1.000
	No	777 (98.9%)	110 (99.1%)	667 (98.8%)	
Thyroid disorder	Yes	52 (6.6%)	4 (3.6%)	48 (7.1%)	0.217
	No	734 (93.4%)	107 (96.4%)	627 (92.9%)	
Urological disease	Yes	5 (.6%)	3 (2.7%)	2 (.3%)	**0.022**
	No	781 (99.4%)	108 (97.3%)	673 (99.7%)	
Unknown	Yes	1 (0.1%)	0 (0.0%)	1 (0.1%)	1.000
	No	785 (99.9%)	111 (100.0%)	674 (99.9%)	
None	Yes	89 (11.3%)	19 (17.1%)	70 (10.4%)	**0.038**
	No	697 (88.7%)	92 (82.9%)	605 (89.6%)	
Smoker	Yes	484 (61.6%)	73 (65.8%)	411 (60.9%)	0.328
	No	302 (38.4%)	38 (34.2%)	264 (39.1%)	
Alcohol	Yes	53 (6.7%)	15 (13.5%)	38 (5.6%)	**0.002**
	No	733 (93.3%)	96 (86.5%)	637 (94.4%)	
H. pylori organisms seen?	Yes	233 (29.6%)	44 (39.6%)	189 (28.0%)	**0.013**
	No	553 (70.4%)	67 (60.4%)	486 (72.0%)	
