# Supplementary material for: Prevalence, Risk Factors, and Endoscopic Findings of Helicobacter pylori Infection Among Lebanese Patients Undergoing Gastroscopy: A Retrospective Study from a Single Tertiary Center
Source: Antibiotics (Basel). 2025 Oct 11;14(10):1013. doi: 10.3390/antibiotics14101013 (PMC12561384; doi:10.3390/antibiotics14101013)
Supplement: Supplementary file 1 [file antibiotics-14-01013-s001.zip › Table_S9.pdf]

Table S9: Percent distribution and univariate analysis of factors associated with non-erosive duodenitis

		Non-erosive Duodenitis			Univariate analysis
		Overall n=786	Yes n=20	No n=766	P-value
Age (Mean±Std)		43.15±13.4	43.25±11.8	43.14±13.5	0.972
Gender	Male	315 (40.1%)	9 (45.0%)	306 (39.9%)	0.649
	Female	471 (59.9%)	11 (55.0%)	460 (60.1%)	
Body mass index (kg/m^2)	Underweight (< 18.5)	36 (4.6%)	0 (0.0%)	36 (4.7%)	0.308
	Normal weight (18.5 - 24.9)	361 (45.9%)	6 (30.0%)	355 (46.3%)	
	Overweight (25.0 - 29.9)	252 (32.1%)	9 (45.0%)	243 (31.7%)	
	Obese (≥ 30)	137 (17.4%)	5 (25.0%)	132 (17.2%)	
Anemia	Yes	22 (2.8%)	0 (0.0%)	22 (2.9%)	1.000
	No	764 (97.2%)	20 (100.0%)	744 (97.1%)	
Autoimmune disease	Yes	1 (.1%)	0 (0.0%)	1 (.1%)	1.000
	No	785 (99.9%)	20 (100.0%)	765 (99.9%)	
Bone disease	Yes	3 (.4%)	0 (0.0%)	3 (.4%)	1.000
	No	783 (99.6%)	20 (100.0%)	763 (99.6%)	
Cancer	Yes	15 (1.9%)	0 (0.0%)	15 (2.0%)	1.000
	No	771 (98.1%)	20 (100.0%)	751 (98.0%)	
Crohn's disease	Yes	6 (.8%)	0 (0.0%)	6 (.8%)	1.000
	No	780 (99.2%)	20 (100.0%)	760 (99.2%)	
Diabetes	Yes	82 (10.4%)	2 (10.0%)	80 (10.4%)	1.000
	No	704 (89.6%)	18 (90.0%)	686 (89.6%)	
Dyslipidemia	Yes	37 (4.7%)	0 (0.0%)	37 (4.8%)	0.618
	No	749 (95.3%)	20 (100.0%)	729 (95.2%)	
Familial Mediterranean fever (FMF)	Yes	3 (.4%)	0 (0.0%)	3 (.4%)	1.000
	No	783 (99.6%)	20 (100.0%)	763 (99.6%)	
Gastroesophageal reflux disease (GERD)	Yes	127 (16.2%)	4 (20.0%)	123 (16.1%)	0.549
	No	659 (83.8%)	16 (80.0%)	643 (83.9%)	
GI disorder	Yes	626 (79.6%)	16 (80.0%)	610 (79.6%)	1.000
	No	160 (20.4%)	4 (20.0%)	156 (20.4%)	
Heart disease	Yes	55 (7.0%)	1 (5.0%)	54 (7.0%)	1.000
	No	731 (93.0%)	19 (95.0%)	712 (93.0%)	
Hemorrhoids	Yes	1 (.1%)	0 (0.0%)	1 (.1%)	1.000
	No	785 (99.9%)	20 (100.0%)	765 (99.9%)	
Hypertension	Yes	152 (19.3%)	1 (5.0%)	151 (19.7%)	0.148
	No	634 (80.7%)	19 (95.0%)	615 (80.3%)	

Irritable bowel syndrome (IBS)	Yes	1 (.1%)	0 (0.0%)	1 (.1%)	1.000
	No	785 (99.9%)	20 (100.0%)	765 (99.9%)	
Kidney disease	Yes	7 (.9%)	1 (5.0%)	6 (.8%)	0.166
	No	779 (99.1%)	19 (95.0%)	760 (99.2%)	
Migraine	Yes	6 (.8%)	0 (0.0%)	6 (.8%)	1.000
	No	780 (99.2%)	20 (100.0%)	760 (99.2%)	
Neurological disease	Yes	18 (2.3%)	0 (0.0%)	18 (2.3%)	1.000
	No	768 (97.7%)	20 (100.0%)	748 (97.7%)	
Polycystic ovary syndrome (PCOS)	Yes	1 (.1%)	0 (0.0%)	1 (.1%)	1.000
	No	785 (99.9%)	20 (100.0%)	765 (99.9%)	
Peutz–Jeghers syndrome	Yes	0 (0.0%)	0 (0.0%)	0 (0.0%)	-
	No	786 (100.0%)	20 (100.0%)	766 (100.0%)	
Psoriasis	Yes	1 (.1%)	0 (0.0%)	1 (.1%)	1.000
	No	785 (99.9%)	20 (100.0%)	765 (99.9%)	
Psychiatric disorder	Yes	4 (.5%)	0 (0.0%)	4 (.5%)	1.000
	No	782 (99.5%)	20 (100.0%)	762 (99.5%)	
Respiratory disease	Yes	24 (3.1%)	1 (5.0%)	23 (3.0%)	0.466
	No	762 (96.9%)	19 (95.0%)	743 (97.0%)	
Rheumatological disease	Yes	9 (1.1%)	0 (0.0%)	9 (1.2%)	1.000
	No	777 (98.9%)	20 (100.0%)	757 (98.8%)	
Thyroid disorder	Yes	52 (6.6%)	0 (0.0%)	52 (6.8%)	0.636
	No	734 (93.4%)	20 (100.0%)	714 (93.2%)	
Urological disease	Yes	5 (.6%)	0 (0.0%)	5 (.7%)	1.000
	No	781 (99.4%)	20 (100.0%)	761 (99.3%)	
Unknown	Yes	1 (0.1%)	0 (0.0%)	1 (0.1%)	1.000
	No	785 (99.9%)	20 (100.0%)	765 (99.9%)	
None	Yes	89 (11.3%)	3 (15.0%)	86 (11.2%)	0.487
	No	697 (88.7%)	17 (85.0%)	680 (88.8%)	
Smoker	Yes	484 (61.6%)	14 (70.0%)	470 (61.4%)	0.433
	No	302 (38.4%)	6 (30.0%)	296 (38.6%)	
Alcohol	Yes	53 (6.7%)	1 (5.0%)	52 (6.8%)	1.000
	No	733 (93.3%)	19 (95.0%)	714 (93.2%)	
H. pylori organisms seen?	Yes	233 (29.6%)	10 (50.0%)	223 (29.1%)	**0.043**
	No	553 (70.4%)	10 (50.0%)	543 70.9%)	
