# Supplementary figures and images for: Prevalence, Risk Factors, and Endoscopic Findings of Helicobacter pylori Infection Among Lebanese Patients Undergoing Gastroscopy: A Retrospective Study from a Single Tertiary Center
Source: Antibiotics (Basel). 2025 Oct 11;14(10):1013. doi: 10.3390/antibiotics14101013 (PMC12561384; doi:10.3390/antibiotics14101013)

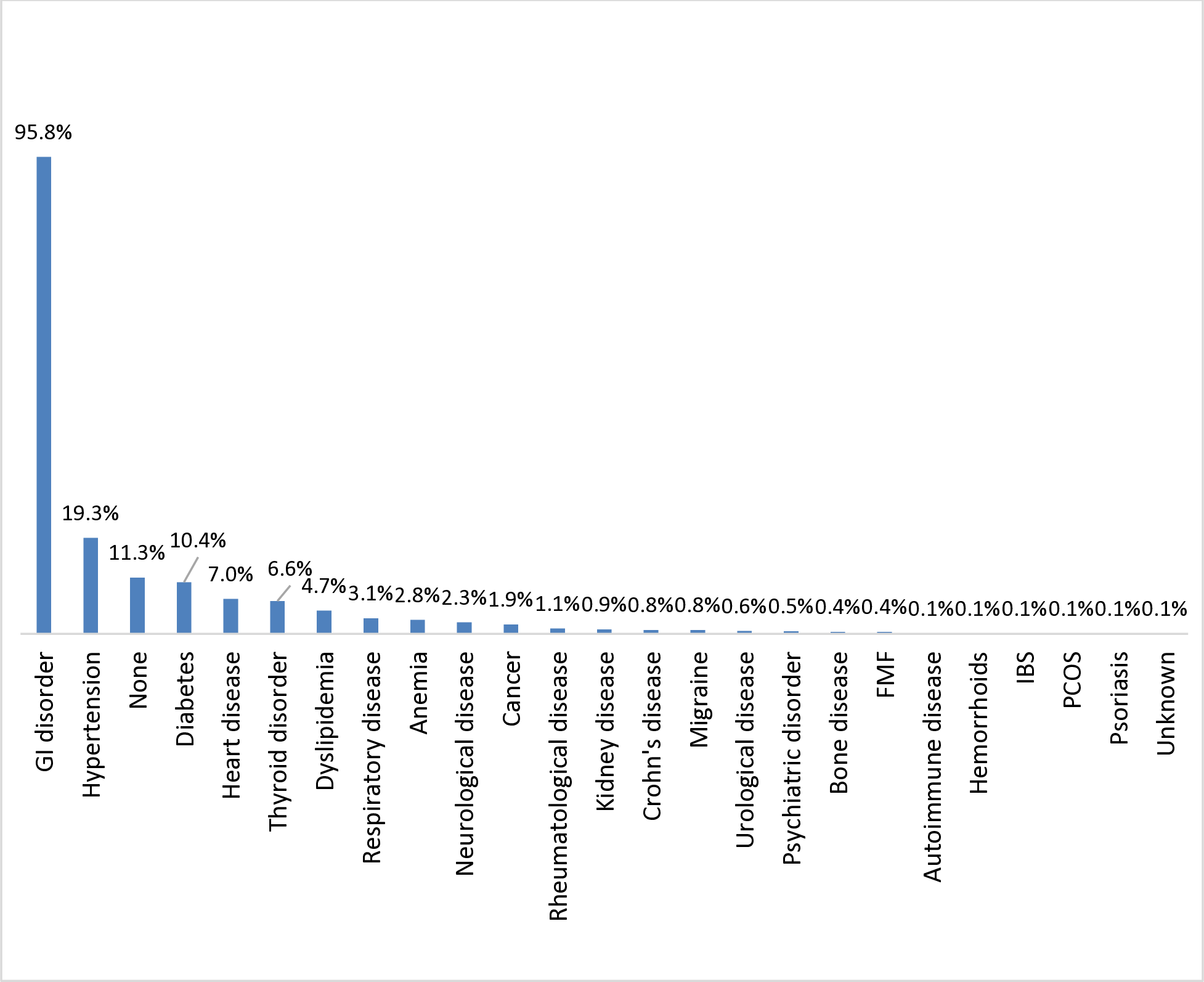

Supplement: Supplementary file 1 [file antibiotics-14-01013-s001.zip › Figure_S1.tif]

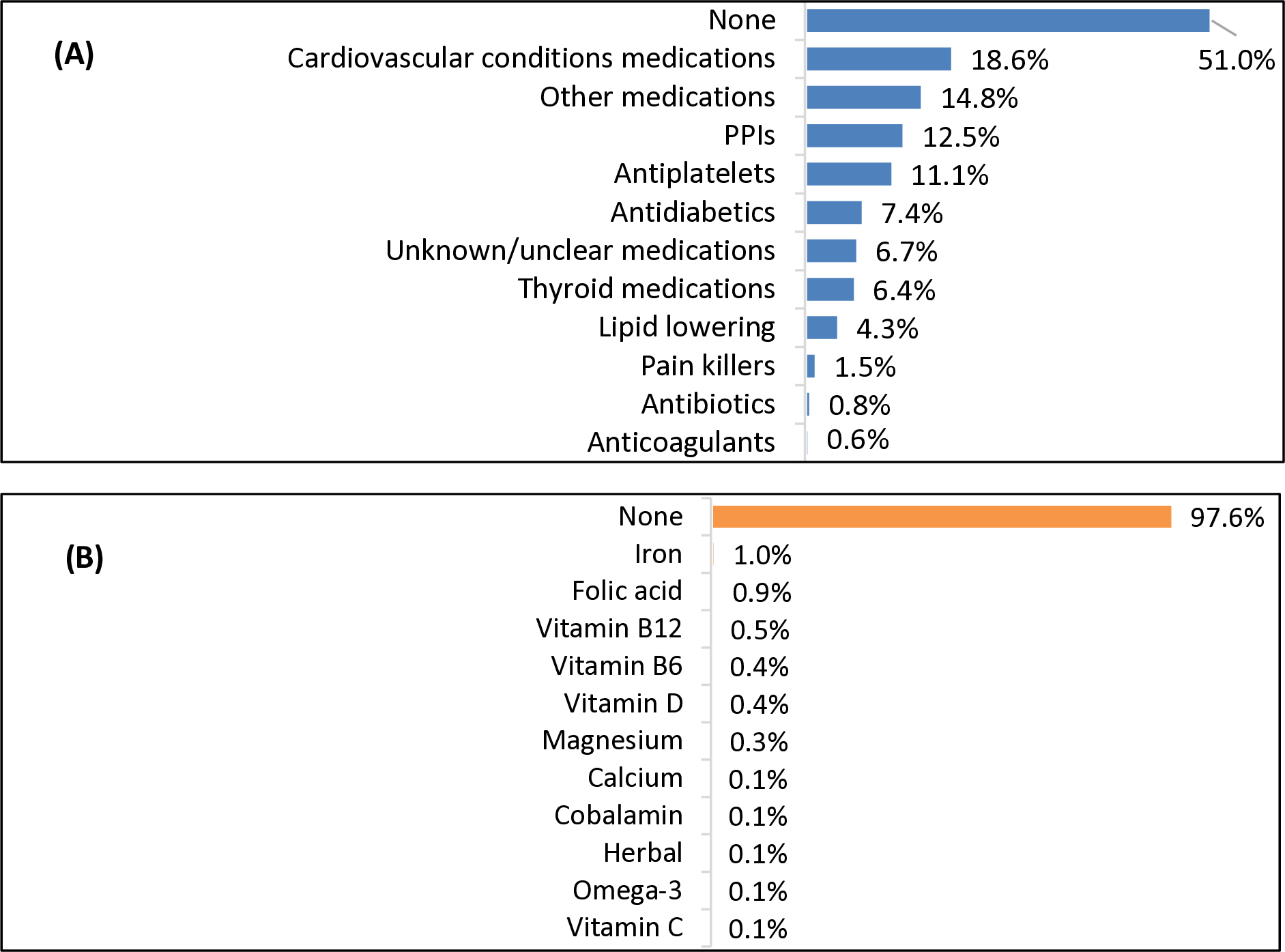

Supplement: Supplementary file 1 [file antibiotics-14-01013-s001.zip › Figure_S2.tif]
